# Hydro-Seq enables contamination-free high-throughput single-cell RNA-sequencing for circulating tumor cells

**DOI:** 10.1038/s41467-019-10122-2

**Published:** 2019-05-15

**Authors:** Yu-Heng Cheng, Yu-Chih Chen, Eric Lin, Riley Brien, Seungwon Jung, Yu-Ting Chen, Woncheol Lee, Zhijian Hao, Saswat Sahoo, Hyun Min Kang, Jason Cong, Monika Burness, Sunitha Nagrath, Max S. Wicha, Euisik Yoon

**Affiliations:** 1 Department of Electrical Engineering and Computer Science, University of Michigan, 1301 Beal Avenue, Ann Arbor, MI 48109-2122 USA; 2 Forbes Institute for Cancer Discovery, University of Michigan, 2800 Plymouth Rd., Ann Arbor, MI 48109 USA; 3 Department of Chemical Engineering, University of Michigan, 2300 Hayward St, Ann Arbor, MI 48109 USA; 4 Computer Science Department UCLA, Boelter Hall, Los Angeles, CA 90095-1596 USA; 5 Department of Biomedical Engineering, University of Michigan, 2200 Bonisteel, Blvd., Ann Arbor, MI 48109-2099 USA; 6 School of Public Health, University of Michigan, 1415 Washington Heights, Ann Arbor, MI 48109-2029 USA; 7 Rogel Cancer Center, University of Michigan, 1500 E. Medical Center Drive, Ann Arbor, MI 48109 USA

**Keywords:** Gene expression analysis, Lab-on-a-chip, Sequencing, Cancer stem cells, Metastasis

## Abstract

Molecular analysis of circulating tumor cells (CTCs) at single-cell resolution offers great promise for cancer diagnostics and therapeutics from simple liquid biopsy. Recent development of massively parallel single-cell RNA-sequencing (scRNA-seq) provides a powerful method to resolve the cellular heterogeneity from gene expression and pathway regulation analysis. However, the scarcity of CTCs and the massive contamination of blood cells limit the utility of currently available technologies. Here, we present Hydro-Seq, a scalable hydrodynamic scRNA-seq barcoding technique, for high-throughput CTC analysis. High cell-capture efficiency and contamination removal capability of Hydro-Seq enables successful scRNA-seq of 666 CTCs from 21 breast cancer patient samples at high throughput. We identify breast cancer drug targets for hormone and targeted therapies and tracked individual cells that express markers of cancer stem cells (CSCs) as well as of epithelial/mesenchymal cell state transitions. Transcriptome analysis of these cells provides insights into monitoring target therapeutics and processes underlying tumor metastasis.

## Abstract (web summary)

Transcriptome analysis of circulating tumor cells (CTCs) provides insights into monitoring target therapeutics and underlying tumor metastasis. Here the authors present Hydro-Seq, a contamination-free high-throughput hydrodynamic scRNA-seq barcoding technique for rare CTCs.

## Introduction

Circulating tumor cells (CTCs) are the rare metastatic cancer cells shed from the primary tumor into the circulatory system as the seeds to form secondary tumors at distant tissues^1,2^. A strong heterogeneity of CTCs is observed in the metastatic process with the cancer cell transition between epithelial and mesenchymal cell types^3,4^. As such, single-cell analysis of those CTCs provides critical insights into cancer metastasis. The access to tumor cells from blood draw or liquid biopsy represents a promising alternative to tumor biopsies for tumor molecular heterogeneity characterization as a companion diagnostic tool^5^. However, such analysis is challenged by the scarcity of CTCs and blood contamination in the samples. In average, less than 100 CTCs are found per 10 mL of blood with billions of erythrocytes and millions of leukocytes^6,7^. To overcome such challenges, substantial research has been focused on enriching CTCs for downstream analysis^1,2,7^. Immunoaffinity enrichment methods can extract the CTCs by their surface membrane markers such as EpCAM for CTC numeration and downstream analysis, but this molecular-marker-based selection biases the sampling population and thus limits the molecular heterogeneity for comprehensive analysis. Physical property enrichment methods such as size-based selection can separate larger CTCs from smaller erythrocytes and leukocytes without limiting to certain molecular markers for selection. However, the enriched cell suspension usually still contains a large number of background cells (erythrocytes and leukocytes), making it difficult for downstream analysis. Although fluorescent staining with immunohistochemistry (IHC), in situ hybridization (ISH), and single-cell western blotting may be applied to study the protein and gene expression of CTCs, the bandwidth constraint imposed by fluorescent imaging limits the number of molecular markers to be studied in the assays^8–11^.

To fully characterize the whole transcriptome profile and pathway regulation in metastatic CTCs, single-cell RNA-sequencing (scRNA-seq) has been applied to few CTCs picked by capillary suction and dielectrophoretic microfluidics with the cells labelled by fluorescent staining^2,12–14^. Nevertheless, these techniques are constrained by their limited throughput and reliance upon antibody-based marker expression. Commonly utilized markers such as EpCAM and cytokeratins may miss a substantial population of CTCs, which do not express these proteins. As such, there is still an unmet need to achieve high-throughput contamination-free scRNA-seq to study the pathway regulation and heterogeneity of CTCs.

Recent advances of massively parallel microfluidics in scRNA-seq have enabled high-throughput analysis of cellular heterogeneity and identification of cellular types by their gene signatures^15–18^. By pairing single barcode beads with single cells in droplets or micro-wells, mRNA molecules from a single cell can be uniquely labelled by a barcode and identified using single-cell whole-transcriptome sequencing. However, the low efficiency of droplet-based technologies such as Drop-seq has limited the applicability in analysis of rare cell populations presented in CTCs. Due to the inefficiency of bead-cell pairing, thousands of cells are required to achieve accurate cell readout. Although methods using micro-wells or hydrogel barcoded beads may achieve a better single-cell capture efficiency, the fidelity of single-cell gene sequencing is still challenged by the massive contamination of erythrocytes and leukocytes even after enrichment^16–18^. Thus, the development of a high-throughput technology capable of efficiently capturing and molecularly interrogating CTCs at single-cell resolution would have significant clinical utility for treatment selection and therapeutic monitoring.

Here, we present Hydro-Seq, a high-efficiency-cell-capture contamination-free scRNA-seq platform, for gene expression profiling of CTCs. Hydro-Seq utilizes size-based single-cell capture to prevent the bias that can be caused by molecular CTC selection. This cell capture scheme achieves a high cell capture efficiency (72.85 ± 2.64%, ± represents standard deviation *n* = 3) to analyze the small number of CTCs in 10 mL of blood samples. To achieve contamination-free single-cell sequencing, the Hydro-Seq chamber incorporates pneumatic valves to enable on-chip washing for cellular and acellular contaminant removal in the chambers. Furthermore, the chamber array can be scaled up to thousands of chambers for massively parallel analysis. We validated the utility of Hydro-Seq by sequencing 666 CTCs from 21 advanced breast cancer patients, identifying cellular heterogeneity in critical biomarkers of tumor metastasis and therapy. Hydro-Seq offers the capability to analyze CTCs with single-cell whole-transcriptome sequencing for metastasis research and companion diagnostics applications.

## Results

### Hydro-Seq CTC single-cell transcriptome analysis method

**Fig. 1:**
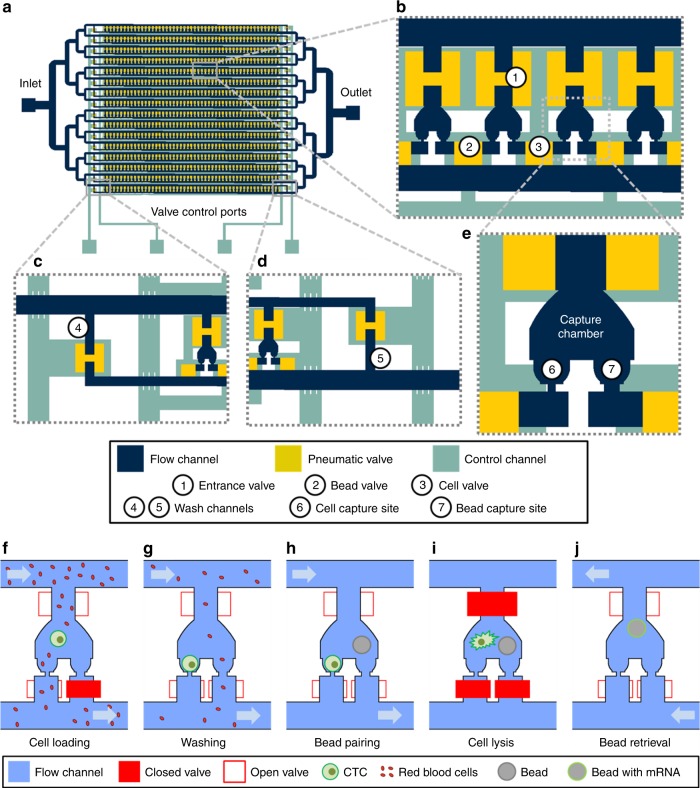
Hydro-Seq, a high capture efficiency scRNA-seq platform for contaminated rare samples. **a**–**e** Design of Hydro-Seq technology. Integrated microfluidic circuit design with valve controls for high-efficiency cell capture and contamination removal. **a** For circulating tumor cells (CTC) application, Hydro-Seq chip is designed with 16 identical branch channels in parallel. Each branch channel consists of 50 chambers for bead-cell pairing, totaling 800 chambers per chip. **b** A closer view highlights the arrangement of parallel chambers. To minimize area consumption, each chamber shares the valve with its neighboring chamber. The entrance valve has a peak height of 45 µm and an area of 200 × 200 µm to enable bead and cell loading. The cell and bead valves are realized in a height of 15 µm and an area of 100 × 100 µm for high-density chamber arrangement. **c**, **d** At the upstream and downstream of branch channels, the valve-controlled wash channels enable channel washing during sample loading and delivery of lysis buffer to the entrance channel for lysis. **e** Enlarged view of a microfluidic bead-cell pairing chamber. **f**–**j** Hydro-Seq operation flow. **f** Bead capture valve is closed during sample loading. The smaller red blood cells flow through the capture sites, while a larger cell (larger than 12 µm) blocks the channel for cell capture. **g** After cell loading, the bead capture valve is then opened to wash the contaminants away. **h** After removing contaminants in the chamber, beads are loaded for pairing. **i** Lysis buffer is introduced into the chamber. After closing all the valves, the beads are moved to the cell capture site for mRNA capture. **j** By opening all the valves and introducing a back flow, the beads can be retrieved to the inlet for downstream preparation

The Hydro-Seq chip consists of four modules (Fig. 1): capture chambers where cells and beads are paired, microfluidic channels through which cells, beads, and lysis buffer are loaded, control valves that selectively close flow paths and isolate the chambers during mRNA extraction, and inlet and outlet ports for introduction of samples and retrieval of beads after RNA extraction. Cells and barcoded beads are hydrodynamically loaded and captured in each capture site and paired in each microfluidic chamber for mRNA barcoding and sequencing (Fig. 1a–e, Supplementary Fig. 1). Each Hydro-Seq chamber contains one cell capture site and one bead capture site in ~1 nL volume, similar to the size of micro-well and droplet volume reported in other scRNA-seq methods. As CTCs are typically larger than other blood cells, the cell capture site was designed with an opening of 10 × 10 µm^1^. This capturing hole allows smaller leukocytes, erythrocytes, and platelets to pass through, while larger cells (CTC or larger leukocytes) can be captured (Fig. 1f, g). The bead capture site was designed with a bowl-shape capture pocket of an opening of 20 × 25 µm to trap the beads with an average diameter of 40 µm (Fig. 1h). The entrance to the chambers, cell capture sites, and bead capture sites can be selectively closed by different Quake valves controlled by pneumatics for the bead-cell-pairing operation. We engineered Hydro-Seq chips composed of 800 chambers per chip to accommodate CTCs from 10 mL of patient blood. This hydrodynamic capture scheme can be expanded to an array of 12,800 chambers, and was demonstrated in the previous work^19^. In this design, we implement 16 branch channels, each containing 50 cell-capture chambers. To enable cell lysis and washing processes, two extra washing channels were added to the entrance and the exit of each branch channel (Fig. 1c, d). To minimize cell loss during cell loading, a pipette tip is directly inserted into the inlet through a punch hole of 1 mm in diameter formed on the microfluidic chip made of Polydimethylsiloxane (PDMS). To load the reagents, beads, and cells in the suspension, the negative pressure is applied in the outlet by a syringe pump, withdrawing the flow from the outlet.

Before loading CTCs into Hydro-Seq, we performed size-based CTC enrichment, and the enriched CTC sample was then transferred to Hydro-Seq for scRNA-seq preparation^7,20^. Although the enrichment step removes several orders of magnitude of blood cells, there are still significant numbers of residual erythrocytes and leukocytes, which are further eliminated by Hydro-Seq (Supplementary Fig. 2, 3). During cell loading, the bead flow channels and washing channels were blocked, so CTCs could be captured at the cell capture sites in the chambers. When a CTC is captured, it blocks the flow path and the following cells are diverted to other downstream chambers, ensuring that only a single CTC occupies each chamber. After cell loading, the bead capture site was then opened to wash away contaminants remaining in the chamber (Fig. 1g). To further remove the potential contaminating cells in the dead volume near the cell capture site, the captured cells were retrieved with 100 µL of phosphate-buffered saline (PBS) to the inlet, and then repeated the cell loading. Considering the total volume of chambers around 800 nL, this reloading process significantly reduced the concentration of contaminants by two orders of magnitude when increasing the solution volume from sub 1–100 µL. Thus, the remaining contaminating cells in the chamber could be effectively removed by repeating the cell loading from the diluted sample followed by the second wash. After washing the chamber, the barcoded beads were then loaded into the chip to pair with the captured cells (Fig. 1h, Supplementary Fig. 4). To lyse the cells, all chambers were first closed by pneumatic valves, and the lysis buffer was introduced to the branch channels through the washing channels. After opening the valves connected to the chambers, cell lysis buffer was introduced into the chambers by a small leakage flow through bead capture sites. The square shape of the bead capture opening and the round shape of the beads make this flow without any additional valve control. Since the cells can block and seal the capture site, they can stay intact in the dead volume of cell capture site. When closing the valves to isolate the chambers, a turbulence flow in the chamber was generated to lyse the cell at the capture site and the released mRNA could be hybridized with the barcoded bead (Fig. 1i, Supplementary Fig. 5, 6). Finally, after 20 min of incubation, the barcoded beads were retrieved by opening all valves with a back flow (Fig. 1j). The rest of the downstream sequencing procedures, including reverse transcription, amplification, library preparation, and paired-end sequencing, are identical to drop-seq protocols^15^.

To demonstrate the high cell capture efficiency of Hydro-Seq, we performed cell loading tests with ~100 cancer cells spiked into blood samples (from healthy donors) enriched by Celsee systems^20^. After loading the enriched spiked samples, we achieved 90.43 ± 6.08% (*n* = 3) single-cell capture efficiency with the large cancer cells and other contaminating cells in the same chamber. After cell capture, a washing procedure was applied to remove contaminating cells and 89.70 ± 5.06% (*n* = 3) of the captured cells remained. Finally, 89.60 ± 6.39% (*n* = 3) of the remaining cells were successfully paired with a single bead for barcoding mRNAs. Accounting for the losses from cell loading, washing, and bead pairing, 72.85 ± 2.64% (*n* = 3) of the initially loaded cell populations were successfully paired with a barcoded bead on-chip. We also tested loading ~10 cancer cells, and 70.44 ± 21.91% (*n* = 3) of the initially loaded cell populations were successfully paired with a barcoded bead on-chip (Supplementary Table 1). The experiments verify that by using the precisely controlled hydrodynamic capture operation, a small number of single cancer cells can be selectively captured and paired with barcoded beads for scRNA-seq with high purity, high efficiency, and high throughput.

### Breast cancer patient CTC transcriptome analysis

**Fig. 2:**
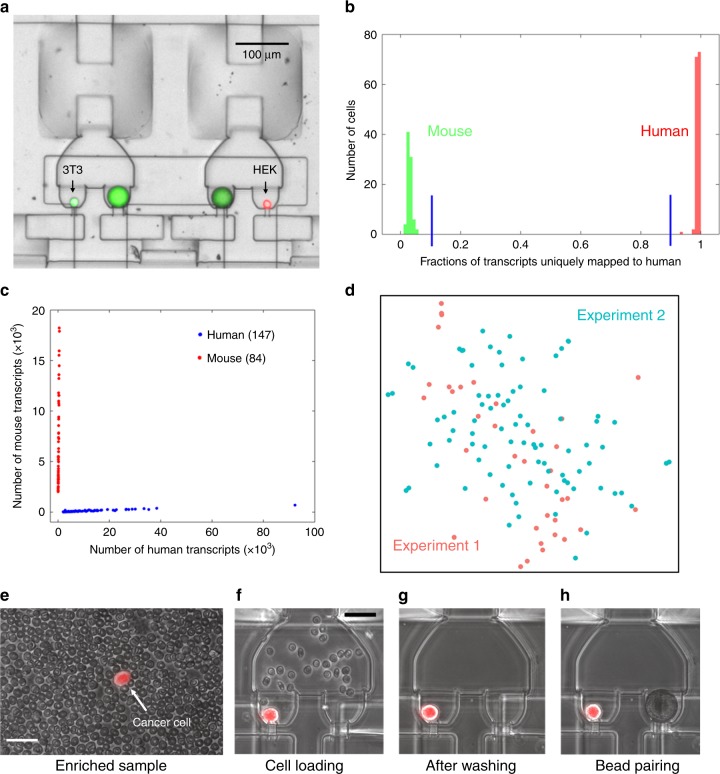
Species-mixing experiment and CTC loading in Hydro-Seq. **a**–**d** Species-mixing experiment. **a** Chambers with beads paired to a mouse cell (3T3 with green fluorescence) and a human cell (HEK with red fluorescence). Fluorescent imaging was applied to examine the pairing condition and verify the capture. **b** Histograms of the percent cross-species contamination of mixed mouse and human cells. Cells with over 90% transcripts mapped to human were labelled red and cells with over 90% transcripts mapped to mouse were labeled green. **c** Species-mixing analysis highlights the single-cell resolution RNA-sequencing with zero cellular cross contamination. Each red dot represents a mouse cell and each blue dot represents a human cell. **d** tSNE plot of single-cell expression analysis for HEK cells from two independent Hydro-Seq experiments. (Experiment 1 labeled with red color and experiment 2 labeled with cyan color) Each dot represents a cell. Cells from two experiments are evenly dispersed and do not show any clustering, indicating good reproducibility of Hydro-Seq. **e**–**h** CTC loading in Hydro-Seq. **e** After CTC enrichment, single-cell RNA-sequencing (scRNA-seq) of the CTCs is still challenged by the presence of many background blood cells. (Scale bar: 25 µm) **f** Erythrocytes flowing through the chamber during sample loading. (Scale bar: 25 µm) **g** During the washing cycle, the bead valve is opened to remove other blood cells through the bead capture flow channel, achieving contamination-free single CTC isolation for bead pairing. **h** Pairing a bead to a single CTC for scRNA-seq. Source data are provided as a Source Data file

Before processing the actual cancer patient blood samples, we first assessed the fidelity of single-cell resolution in mRNA sequencing using Hydro-Seq. We performed a species-mixing experiment using a mixture of human cells (HEK293) and mouse cells (3T3). After bead-cell pairing, we utilized fluorescent imaging to confirm 156 human cells and 80 mouse cells successfully paired with barcoded beads without any instance of two cells from different species in the same chamber (Fig. 2a). In our design, the cell captured in a chamber hydrodynamically prevents the next cell being captured in the same chamber. Even if a cell tailgates after another cell into the chamber, the second cell is removed later during washing cycles. Using shallow sequencing (~60,000 reads per cell), the species-mixing sequencing results demonstrated highly organism-specific libraries without any mixed genotype cells (Fig. 2b, c). Using 800 genes per cell as a threshold, we successfully recovered 147 human and 84 mouse cells, consistent with the number of cells observed by fluorescent microscopy. In addition, the HEK cells processed on different chips showed similar gene expression profiles (Fig. 2d). The species-mixing experiment result shows no cross contamination among the loaded 236 cells as an important attribute of the system. In addition, visual inspection of bead-cell-pairing in the Hydro-Seq chip during the protocol ensures the quality of single-cell RNA-sequencing experiments. These advantages distinguish Hydro-Seq from droplet-based single-cell transcriptome sequencing technologies.

**Fig. 3:**
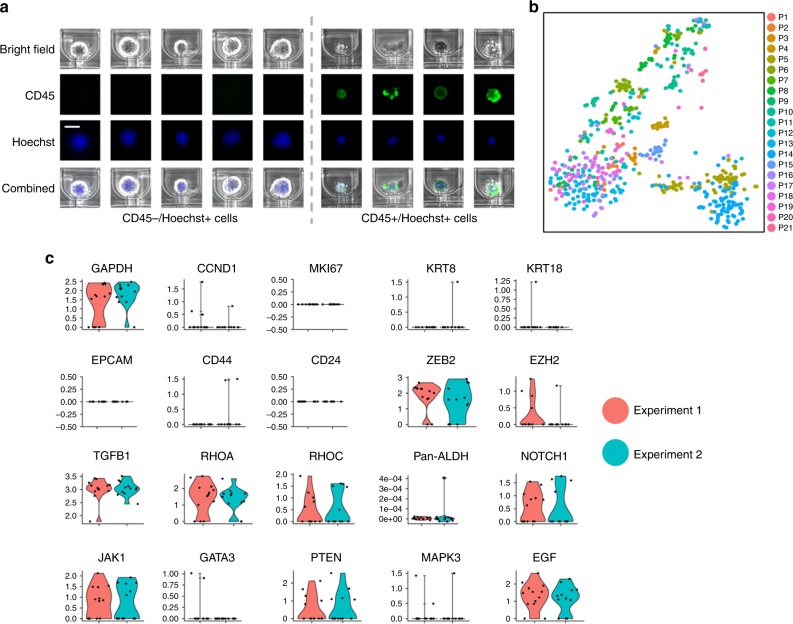
Immunostaining and single-cell RNA-sequencing of CTCs. **a** With CD45 and Hoechst staining, the CD45 positive nucleated cells were identified as leukocytes. (Scale bar: 15 µm) **b** tSNE plot of all CTCs from patient samples processed by Hydro-Seq. (666 CTCs from 21 patient samples with each color representing individual patient sample.) **c** The reproducibility test of Hydro-Seq processing. For the two tubes of blood drawn from the same patient and processed on the same day, comparable CTC counts were achieved from the two experiments (Exp. 1: 13 CTCs with red color; Exp. 2: 12 CTCs with cyan color). The expression profiles of housekeeping, cell proliferation, epithelial, mesenchymal, and other genes are consistent, showing good reproducibility. Source data are provided as a Source Data file

To demonstrate the clinical applicability of Hydro-Seq, we utilized this technology to analyze CTCs obtained from 21 patients with metastatic breast cancer, achieving single-cell transcriptome analysis of 666 CTCs (Supplementary Table 2). Ten milliliter of venous blood samples were first collected from the patients using K2-EDTA tubes. To enrich the CTCs, the samples were processed using the two different size-based CTC enrichment methods including the commercially available Celsee technology and an in-house microfluidic technology dubbed as Labyrinth^7,20^. Although these platforms significantly enriched CTCs, there were still significant cell populations of residual erythrocyte and leukocytes (Fig. 2e). The contaminated cells were further purified in wash cycles (Fig. 2f, g) and paired with beads (Fig. 2h, Supplementary Fig. 7). Some larger leukocytes could be still captured in the capture sites. We applied Hoechst and CD45 staining in the loaded cells to identify the CD45+ leukocytes and the CD45− nucleated cells as the presumed CTCs. (Fig. 3a). After sequencing, gene expression data were analyzed utilizing R-based Seurat kit (Fig. 3b). To ensure high quality contamination-free scRNA-seq data for analysis, the few remaining contaminating leukocytes and erythrocytes were excluded based on the expression of CD45 (PTPRC) or hemoglobin (Supplementary Fig. 8). A low percentage of genes expressed by leukocytes and erythrocytes were detected, demonstrating the capability of Hydro-Seq to successfully screen out the contaminating cell populations. Unhealthy cells were excluded based on the percentage of mitochondria genes. The resulting cells after quality check have at least 800 genes/cell detected from sequencing. To demonstrate the reproducibility of these procedures, we processed two tubes of blood drawn from the same patient on the same day. We detected the comparable number of CTCs (Exp. 1: 13 CTCs, Exp. 2: 12 CTCs) and a similar CTC gene expression profile from these two biological replicates (Fig. 3c).

### Clinical markers identification in CTCs for liquid biopsy

**Fig. 4:**
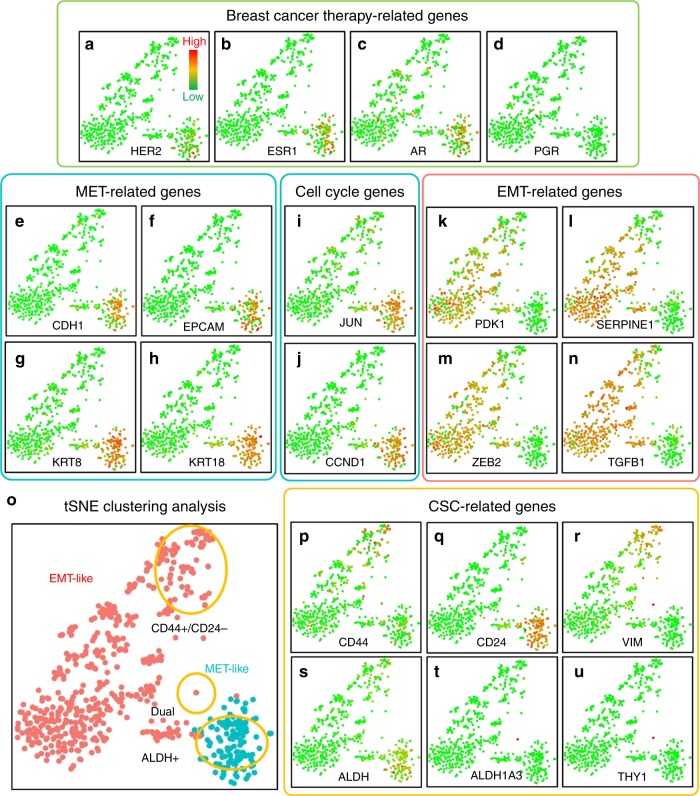
Gene expression and clustering of breast CTCs. **a**–**d** Breast cancer therapy related genes: **a** human epidermal growth factor receptor 2 (HER2/Erbb2), **b** estrogen receptor (ESR1), **c** Androgen (AR), and **d** progesterone receptor (PGR). **e**–**h** MET-related genes: **e** E-cadherin (CDH1), **f** Epithelial Cell Adhesion Molecule (EPCAM), **g** Keratin-8 (KRT8), and **h** Keratin-18 (KRT18). **i**, **j** Cell cycle genes: **i** c-jun (JUN), and **j** cyclin D1 (CCND1). **k**–**n** EMT-related genes: **k** Phosphoinositide-dependent kinase-1 (PDK1), **l** Plasminogen activator inhibitor-1 (SERPINE1), **m** EMT transcription factor (ZEB2), and **n** transforming growth factor β (TGFB1). **o** The clustering and separation of HER2+ MET-like and HER2- EMT-like CTCs. **p**–**u** Stemness related genes: **p** CD44, **q** CD24, **r** vimentin (VIM), **s** pan-ALDH isoforms (ALDH), **t** ALDH1a3, and **u** CD90 (THY1). (Each dot represents one CTC. Green color represents the lowest expression, and red color represents the highest expression. The expression is logarithmically normalized. 666 CTCs from 21 patient samples were plotted based on tSNE clustering method.) Source data are provided as a Source Data file

Current treatments for breast cancer are chosen based on the expression of receptors estrogen (ER), progesterone (PR), as well as human epidermal receptor 2 (HER2) in the initial biopsy^21,22^. However, intra-tumor cellular heterogeneity as well as evolution of tumors over time and following therapy suggests the potential utility of accessing these markers in CTCs. In fact, previous studies have demonstrated the heterogeneity of the expression of these markers in CTCs as well as the discordance between CTCs and primary tumors^3,23^. Utilizing Hydro-Seq, we were able to detect expression of ER, PR, and HER2 in the CTCs (Supplementary Fig. 9). Consistent with previous reports, we observed intra-patient heterogeneity as well as discordant molecular profiles in CTCs compared to the reported phenotype of primary tumors (Fig. 4a–d). However, unlike immunohistochemistry which is limited to assessment of a small number of markers, Hydro-Seq provides the comprehensive dataset on the expression of thousands of genes/cell providing important insights regarding cellular heterogeneity as well as identifying key cell regulatory pathways in these cells.

### Epithelial and mesenchymal state transition of CTCs

When further investigating the molecular characteristics of CTCs, we found they were readily separated by t-distributed stochastic neighbor embedding (tSNE) to two subpopulations: Epithelial (mesenchymal-to-epithelial transition, or MET) cells with HER2+ expression and mesenchymal (epithelial-to-mesenchymal transition, or EMT) cells with HER2- expression (Fig. 4a, and Fig. 4o)^24,25^. The separation is independent of the number of genes detected, the number of transcripts per cell, and the percentage of mitochondrial genes (Supplementary Fig. 10). MET-like CTCs express epithelial markers, including E-cadherin (CDH1), Epithelial Cell Adhesion Molecule (EPCAM), Keratin-8 (KRT8), and Keratin-18 (KRT18) (Fig. 4e–h). In addition, HER2+ MET-like CTCs have been previously reported to be proliferative, consistent with high expression of cyclin D1 (CCND1) and c-jun (JUN) in these cells (Fig. 4i, j)^26^. On the contrary, HER2− EMT-like CTCs express the EMT associated PDK1, SERPINE1, transcription factor ZEB2 as well as transforming growth factor β (TGFB1) (Fig. 4k–n)^27–29^. These results confirm and extend previous studies demonstrating heterogeneous nature of CTCs^4^.

### Cancer stem-like cells within CTCs

It has been previously reported that CTCs are enriched for cells expressing a stem-like phenotype (cancer stem-like cells, or CSCs) (Fig. 4p–u) that have been reported to exist in alternate epithelial or mesenchymal states^23^. The most well-documented epithelial CSC markers in breast cancer are isoforms of aldehyde dehydrogenase (ALDH), while the mesenchymal CSC are characterized as CD44+ and CD24−^30,31^. Consistent with previous reports, ALDH isoforms are over-expressed in the MET-like CTCs (Fig. 4o and Fig. 4s) along with other well-known CSC regulatory genes including such as BMI1, GATA3, and SOX9 (Supplementary Fig. 8). The CD44+/CD24− mesenchymal CSCs expressed the genes associated with EMT, including vimentin (VIM) (Fig. 4o–r). Other known CSC regulatory genes including STAT3, Notch 1, and Notch 2 are also expressed in a sub-fraction of CTCs (Supplementary Fig. 11). From the whole-transcriptome sequencing data, Hydro-Seq enables the elucidation of pathway activation at single-cell resolution. Furthermore, we could detect a small population of CSCs that simultaneously express epithelial (ALDH1A3) and mesenchymal (CD90, or THY1) markers (Fig. 4t–u). The presence of these cells is consistent with recent studies suggesting that CTCs simultaneously expressing epithelial and mesenchymal markers may be endowed with the highest degree of cellular plasticity and metastatic potential^32,33^.

### Intra-patient CTC heterogeneity

**Fig. 5:**
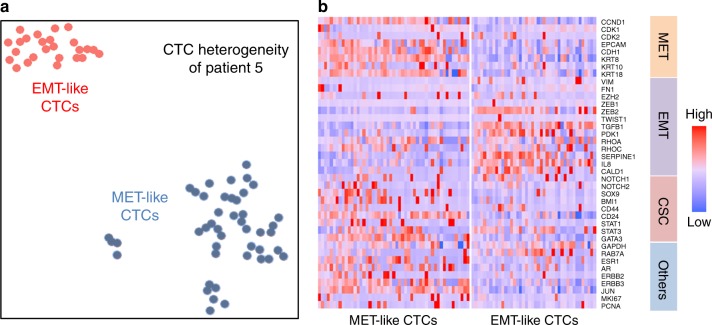
CTC intra-patient heterogeneity of EMT-like and MET-like states. **a** 78 CTCs from the same patient sample were plotted based on tSNE clustering method, demonstrating a clear separation between MET-like and EMT-like CTC populations. **b** Heatmap shows the significant gene expression differences between two populations in EMT, MET, and CSC related genes. Source data are provided as a Source Data file

In addition to substantial inter-patient heterogeneity of transcriptome expression, we also found considerable intra-patient heterogeneity. For example, we detected both HER2+ MET-like and HER2− EMT-like CTCs (Fig. 5) in the same patient with PR+ breast cancer. This intra-patient heterogeneity would be missed by the detection techniques utilizing pooled samples. Although EMT/MET cells could be identified by staining markers, with Hydro-Seq whole-transcriptome sequencing, we can further investigate the difference in pathway regulation between subpopulations, and identify the activation of ITG linked kinase signaling, E-cadherin and Adherens junctions, and FAK kinases involved in EMT/MET state transitions (Table 1)^34,35^. Previous studies have demonstrated that tumor metastasis is dependent on the plasticity of cancer stem-like cells to transition between epithelial and mesenchymal states. Consistent with this concept, we found that the EMT and MET CTCs have distinguishable activation of crucial CSC regulation pathways, including c-Myc, PDGFR, RhoA, telomerase and ERB1 signaling (Table 1)^36–40^. These results demonstrate the potential of Hydro-Seq in downstream transcriptome analysis to provide insights into the biology of CTCs and cancer metastasis.

**Table 1:** Pathways distinguishing the HER2+ MET-like and HER2− EMT-like CTCs. The *p*-value was determined by Fisher’s Exact Test

Top-ranked pathways	*p*-value	Roles
Integrin-linked kinase signaling	2.17E-08	EMT/MET
Stabilization and expansion of the E-cadherin adherens junction	3.66E-06	EMT/MET
Signaling events mediated by focal adhesion kinase	1.21E-04	EMT/MET
Validated targets of C-MYC transcriptional activation	1.38E-04	CSC
PDGFR-beta signaling pathway	6.56E-04	CSC
AP-1 transcription factor network	5.88E-04	
RhoA signaling pathway	3.28E-04	CSC
Regulation of Telomerase	1.73E-03	CSC
ErbB1 downstream signaling	2.22E-03	CSC
PAR1-mediated thrombin signaling events	1.16E-03	

## Discussion

Although previous studies have demonstrated that CTC enumeration provides valuable prognostic information, molecular analysis of these cells offers far greater potential for obtaining biological, and clinical insights^41^. Hydro-Seq achieves the molecular interrogation of CTCs with whole-transcriptome analysis, identifying cellular heterogeneity of important clinical and metastatic biomarkers. Utilizing hydrodynamic cell captures in parallel chambers, Hydro-Seq attains a high cell capture efficiency to isolate the limited CTCs available for analysis (Supplementary Table 3). The bead-cell-pairing chambers with on-chip pneumatic valves enable the removal of contamination and the isolation of chambers during transcript capture. Finally, the chamber array scaling achieves high-throughput analysis of CTCs.

To analyze the molecular heterogeneity of CTCs, fluorescent staining with ISH and IHC remains as the conventional way to investigate CTC gene and protein expressions. To identify the CTCs and exclude the leukocytes, the cells are typically stained with a nucleus marker (DAPI or Hoechst), CD45, and pan-cytokeratin (Pan-CK), where CTCs are usually the nucleated and Pan-CK-positive cells and leukocytes are nucleated and CD45-positive cells. Due to the bandwidth limitation in fluorescence imaging, multiplex fluorescent assay can only achieve up to 6–7 channels (or markers) at a time. Considering the three markers used for CTC identification, only 3–4 channels are available for studies. Although it is possible to remove the antibodies and apply new ones for staining, the procedure is repetitive and difficult to scale up^42,43^. Alternate way of analyzing the whole-transcriptome profile of CTCs is to use serial cell picking to isolate single cells in individual tubes for scRNA-seq^2,12^. The CTCs are usually first identified by EpCAM or pan-cytokeratin staining. Single-cell manipulation methods such as capillary suction and dielectrophoretic microfluidics are then applied to dispense target cells to different tubes for downstream processing. Although this methodology has successfully achieved scRNA-seq of CTCs, the serial picking process is laborious, low throughput, and difficult to scale up the number of cells to analyze from each sample, limiting its application for CTC heterogeneity studies. Furthermore, the molecular selection of EpCAM and pan-cytokeratin can miss important CTC subpopulations such as the mesenchymal CTCs presented in this paper. Therefore, there is a trade-off between the number of cells and genes for CTC studies using these conventional methods. Fluorescent ISH and IHC assay achieves high-throughput analysis of CTCs but with a limited number of biomarkers available for analysis, preventing the study of pathway regulation for treatment development. Cell picking with scRNA-seq enables whole-transcriptome profiling of CTCs but with a limited number of cells for analysis, limiting the capability to study the cellular heterogeneity, which is a critical characteristic of tumor cells. To overcome such limitation, Hydro-Seq achieves both high-throughput and whole-transcriptome analysis of CTCs to access cellular heterogeneity as well as to elucidate cell regulatory pathways. There are other massive-parallel microfluidic techniques for scRNA-seq, such as commercially available Fluidigm C1 system, DEPArray, Drop-seq, etc. But these techniques cannot provide a high single-cell capture efficiency for rare cells. The other challenge to overcome is the massive contamination of residual erythrocytes in the size-based CTC enriched samples. The inability to further remove the contamination is detrimental to achieve high-fidelity single-cell whole transcriptome sequencing results.

CTC analysis represents a promising alternative to analyze the molecular property of cancer cells for treatment selection and patient monitoring with the much less invasive venous blood draw. Conventionally, physicians often need to collect the tissue samples by invasive surgery or tumor biopsy for molecular tests. In this study, we demonstrated that drug targets for breast cancer including ER, PR, AR, and HER2 were successfully detected from the CTCs, highlighting the potential use of Hydro-Seq for precision medicine applications. To further validate the value of Hydro-Seq as a companion diagnostic tool, new clinical trials can be initiated to study and compare the tumor profile between CTCs and biopsy samples at the same time. Furthermore, the ability to readily obtain these cells over time allows for assessment of treatment effects as well as elucidating mechanisms of treatment resistance. This may aid in treatment selection and patient monitoring. In this context, Hydro-Seq provides an agnostic companion diagnostic across a wide spectrum of cancer clinical trials.

Since CTCs represent the metastatic tumor cells in the circulatory system, scRNA-seq analysis of those cells also provides insights into the molecular regulation during cancer metastasis. It was previously reported that cancer cells perform the transitions between epithelial and mesenchymal cell types for tumor metastasis^27,44–46^. In the primary tumor, the epithelial type cells are proliferative and cycling to reproduce cancer cells for tumor growth. To acquire the increased motility and invade the stroma, some cancer cells can perform the EMT to acquire the mesenchymal cell property for cell migration and intravasation into circulatory systems. These mesenchymal cells are also quiescent, capable of staying dormancy and surviving the harsh environment during circulation. To form a secondary tumor, some CTCs can perform the extravasation to a distant tissue and MET to restore the epithelial cell property, allowing the cells to proliferate again for colonization. In our sequencing results, the unsupervised learning tSNE algorithm was able to cluster the CTCs into two populations with each expressing EMT-related and MET-related genes, consistent with previous literature on metastasis^47^. Interestingly, most of the CTCs analyzed in this study were mesenchymal cells, which would be missed using molecular CTC enrichment methods^48,49^. Furthermore, we were able to identify a small population of cells expressing the stemness markers representing both epithelial CSCs (ALDH) and mesenchymal CSCs (CD44+/CD24−)^30,50–52^. These cells exhibit the cancer plasticity to perform EMT/MET and further differentiate into regular tumor cells. The results have validated the utility of Hydro-Seq to study metastasis. Examination of other genes expressed in these cell populations provides further insights into their regulatory pathways.

In summary, we have developed Hydro-Seq, a platform, which enables single-cell transcriptome analysis of rare and limited cell populations such as CTCs with advantages over existing technologies including: (1) Size-based CTC isolation prevents the bias caused by marker-based isolation and enables observing heterogeneity of CTCs; (2) High-efficiency and high-throughput CTC profiling facilitates the discovery of rare CTC subtypes; and (3) Whole-transcriptome sequencing of CTCs provides a comprehensive transcriptome analysis compared to the limited fluorescent staining techniques. We demonstrated the capability of Hydro-Seq technology by performing single-cell transcriptome analysis of 666 CTCs obtained from 21 patient samples. While there are a number of CTC enrichment technologies that have been developed and commercialized, Hydro-Seq provides a much more advanced downstream analysis capability for molecular characterization of CTCs at single-cell resolution beyond CTC enrichment. This should be of great utility in therapeutic selection, monitoring of cancer patients, and fundamental understanding of metastasis and cellular heterogeneity of CTCs.

## Methods

### Device fabrication

The devices were made using soft lithography fabrication processes. The multi-layer layout of the chip was designed using AutoCAD 2016 (Autodesk^®^). The masks for photolithography were made using a mask making instrument (μPG 101, Heidelberg instruments). The mold for the flow channel was fabricated with 10, 20, 40, and 100 µm thick SU-8 (Microchem) following the manufacturer’s protocol. The valves were created using AZ^®^9260 (AZ Electronic Materials) with a peak thickness of 15 µm and 45 µm, respectively, after thermal reflow at 150 °C overnight to create a curved structure. The mold for the control channels was fabricated with 20 µm SU-8. The SU-8 mold was treated by vaporized Trichloro (1 H,1 H,2 H,2H-perfluorooctyl) Silane (448931 Aldrich) under vacuum overnight to promote the release of cured PDMS. After coating, the mold was heated at 150 °C on a hot plate for 10 min. PDMS (Sylgard 184, Dow Corning) was prepared by mixing with 10 (elastomer): 1(curing agent) (w/w) ratio, poured on the flow channel molds, and cured at 85 °C overnight before peeling. A thin film of PDMS was spun onto the control channel mold and cured at 85 °C for 1 h. After peeling the PDMS from the flow control mold, the PDMS piece and the thin film PDMS were treated using oxygen plasma (80 W for 60 s) and bonded using MJB3 aligner (Karl Suss). The devices after bonding were heated at 80 °C overnight to ensure bonding quality.

### Cell culture

Different cell lines, including MDA-MB-231 (ATCC^®^ HTB-26™), MDA-MB-231 GFP, HEK293 (ATCC^®^ CRL-1573™), and 3T3 (ATCC^®^ CRL-1658™) were cultured in petri dishes for device testing. MDA-MB-231 cells were obtained from Dr. Gary Luker’s Lab (University of Michigan, MI, USA). MDA-MB-231 GFP cells were obtained from Dr. Celina Kleer’s Lab (University of Michigan, MI, USA). HEK293 and 3T3 cells were obtained from Dr. Max Wicha’s Lab (University of Michigan, MI, USA). The cell lines were authenticated and regularly checked to exclude mycoplasma contamination in those labs. MDA-MB-231, MDA-MB-231 GFP, HEK293 and 3T3 cells were cultured in DMEM (Gibco 11965) with 10% FBS (Gibco 10082) and 1% penicillin/streptomycin (Gibco 15070). All the cells were cultured and passaged when cells reached over 80% confluency in the dish.

### Image acquisition

The microfluidic devices were imaged using an inverted microscope (Nikon) with a XYZ motorized stage (ProScan II, Prior Scientific). The bright-field and fluorescent images were taken with a ×4 objective and a ×10 objective, respectively, with a charge-coupled device (CCD) camera (Coolsnap HQ2, Photometrics). A FITC, a TRITC, and a UV-2A filter cube were used for the fluorescent imaging. To ensure optimized image quality, auto-focusing was done after imaging every 5 frames. After scanning, the Nikon NIS-Elements Basic Research software module was used to stitch individual images to a large image for analysis.

### Sealing test

Before testing, the device was placed in a desiccator with vacuum pressure for 20 min. After the vacuum process, the device was primed using a 5% (w/w) PEO-terminated triblock polymer (Pluronic^®^ F108, BASF) in DI water for 20 min. The small-molecule dye solution (fluorescein 5(6)-isothiocyanate, F3651, Sigma–Aldrich, molecular weight of 389 Daltons) was used to test the leakage of valves in Hydro-Seq chips. After flowing PBS through the chip, all chamber valves were closed, and dye solution was introduced to the branch channel. The chip was then imaged at 0, 10, and 20 min after dye introduction to test the sealing of chambers.

### Hydro-Seq device priming and preparation

For scRNA-sequencing, the device was heated at 150 °C for 30 min. The device was then placed in a desiccator with vacuum pressure for 20 min. After the vacuum process, the device was sanitized using UV radiation and primed using a 5% (w/w) PEO-terminated triblock polymer (Pluronic® F108, BASF) in DI water for 20 min. Before cell loading, channels were washed by flowing through 150 µL phosphate-buffered saline (PBS). To enable pneumatic valve control, the tubing filled with DI water was then connected to the designated ports with a back pressure of 25PSI when activated. The syringe pump was connected to the outlet of the chip to drive cell loading process.

### Cell capture efficiency test

MDA-MB-231 cells were first stained by green CellTracker dye (ThermoFisher C2925) with 10 µM concentration following manufacturer’s protocol and then suspended using Trypsin–EDTA (Gibco 25200). Cell concentrations were first calculated using a hemocytometer, and cells were diluted to a concentration of 500 cells/mL. The suspended cells were sampled three times by loading 100 µL of the solution to a well in a 96-well plate. Each well was imaged to count the number of cells and verify that each 100 µL solution contains 50 cells in average. After sampling, another 100 µL cell solution was then loaded to the Hydro-Seq chip by directly inserting the pipette tip to the inlet. The solution was then loaded with a flow rate of 10 µL/min until emptying the pipette tip. During cell loading, bead valves and wash channels were closed to prevent cells from passing by. After loading the cells, the pipette tip was then removed and another 20 µL PBS was added to the inlet and loaded to ensure all the cells in the inlet and branching channels entered the cell-capture chamber array. The number of captured cells was then quantified by large-area fluorescent imaging. The washing protocol was applied to the captured cells. During the washing cycle, the bead valves were opened and 100 µL of PBS was flushed with a flow rate of 50 µL/min from the inlet to the outlet. After flushing, the bead valves were closed again. Then, 100 µL of fresh PBS was added to the outlet and withdrawn from the outlet to the inlet by a pipette. The withdrawn 100 µL solution was then reloaded back to the chip again with a flow rate of 10 µL/min. The chip was imaged again using fluorescent microscopy to evaluate the number of captured cells after washing. Finally, the beads were loaded with a 150 µL bead solution with 10k beads/mL concentration at a flow rate of 50 µL/min. During the bead loading, only the wash channels at the upstream branch channels remained closed. After bead loading, the chip was imaged again using fluorescent microscopy to quantify the number of bead-cell pairs.

### Cell capture efficiency test of the enriched spike samples

MDA-MB-231 cells were first stained by green CellTracker dye (ThermoFisher C2925) with 10 µM concentration following manufacturer’s protocol and then suspended using Trypsin–EDTA (Gibco 25200). Cell concentrations were first calculated using a hemocytometer, and cells were diluted to a concentration of 2000 cells/mL. one hundred microliter of the cell solution was then spiked to 7 mL of whole blood from healthy donors. The blood sample was then processed by Celsee following manufacturer’s protocol (https://www.celsee.com/). The enriched CTC sample was then spun down to 200 µL from 4 mL. The solution was then pipetted up and down gently for mixing. After mixing, the first 100 µL solution was used for cell loading following the protocol in cell capture efficiency test. The second 100 µL solution was then taken to a well in 96-well plate to quantify the number of cells in the enriched sample. The well was scanned after 30 min to ensure cells precipitate to the bottom of the well for fluorescent imaging.

### Species-mixing experiment

HEK293 cells were stained with CellTracker Red (ThermoFisher C34552), and 3T3 cells were stained with CellTracker Green (ThermoFisher C2925) following manufacturer’s protocol. HEK293 and 3T3 cells were suspended using Trypsin–EDTA (Gibco 25200) and diluted into 25k cells/mL in PBS. The cell suspension solution was then loaded into the device inlet and cells were driven into the chip by syringe pump with a flow rate of 10 µL/min. The valves in the bead capture sites were closed during cell loading. After cell loading, all valves were closed and the residual cells in the inlet and channels were washed away using PBS. Beads stored in TE buffer were then re-suspended in PBS twice and loaded into the chip with 20k beads/mL by gravity flow. During the loading, all the chamber valves were opened to enable bead-cell pairing inside the chambers while wash channels remained closed. Since the beads settle down easily in the inlet, it was required to pipette up and down during the loading process to redistribute beads in the suspension. After bead-cell pairing, the chamber valves were again closed, and wash channels were opened to allow cell lysis buffer to flow into the branch channel. Then, the chamber valves were opened for 5 s to allow lysis buffer to enter the chamber for cell lysis. After lysis, the devices were tilted to move the beads to cell capture site and incubated for 20 min for mRNA capture. Finally, the valves were opened, and beads were retrieved by drawing 200 µL of PBS from the outlet to the inlet using a pipette. The retrieved beads were then processed for sequencing according to the Drop-seq protocol.

### Patient sample CTC sequencing experiments

Whole blood was obtained from adult patients with metastatic breast cancer at the University of Michigan Rogel Cancer Center. The blood collection complied with standard ethical regulations pertaining to work with human subjects; the research protocol and informed consent were approved by the University of Michigan Medical School Institutional Review Board (IRBMED). All subjects were consented by a study team member prior to the blood draw. For experiments with Labyrinth purified samples, the blood samples (~10 mL) were processed using the protocol reported in a prior literature^7^. For experiments with Celsee purified samples, the blood samples (~10 mL) were processed by the Celsee PREP100 system following manufacturer’s protocol. For both technologies, the samples were spun down to 100 µL volume after CTC enrichment. After device priming and preparation, the CTC suspension was loaded to the device by inserting the pipette tip filled with the 100 µL solution to the inlet. After closing all the wash channels and bead valves, the CTC suspension was loaded to the chip with a flow rate of 10 µL/min driven by a syringe pump. After emptying the pipette tip, the tip was removed and 100 µL PBS was added to the inlet. After washing with PBS at 10 µL/min for 2 min, the bead valves were opened, and the flow rate was increased to 50 µL/min to wash away residual red blood cells in the chamber. The PBS solution was refilled during the wash process. After washing for 3 min, the flow was stopped, and bead valves were closed again. To further remove contaminating cells in the chamber, 100 µL PBS was added to the outlet and a pipette tip was inserted to the inlet to retrieve the solution using a pipette. Then, the solution was loaded back to the chip and washed again using the same protocol in the first loading. After loading CTCs, the beads were loaded to the chip and prepared following the same procedure described in the species-mixing session.

### Gene sequencing

We obtained beads from the Hydro-Seq chip and processed the beads following the Drop-seq protocol including RT (using Thermofisher Maxima RT kit), PCR (using Kapa HiFi Hotstart PCR Readymix), and library preparation (using Illumina Nextera XT Library Prep Kit)^17^. The DNA samples were quantified and pooled by the University of Michigan Sequencing Core for sequencing. We pooled 10 samples to be sequenced using a NextSeq 500 mid-output sequencing lane. Each population is expected to have approximately 10 million reads (paired-end: one side 25 base pairs for barcode and the other side 115 base pairs for mRNA quantification).

### Read alignment and data analysis

The sequencing reads were aligned using STAR and processed by Drop-seq_tools-1.12 (https://github.com/broadinstitute/Drop-seq/releases) flow suggested by Drop-seq^17^. Then, gene sequencing data were analyzed using Seurat, an R package for single-cell analysis (http://satijalab.org/seurat/). Cells with more than 800 genes detected were considered to be successfully captured and analyzed. The cells having more than 5% mitochondrial gene expression were discarded for their poor viability. White blood cells (WBCs) were removed by eliminating any barcode with more than 0.01% of CD45 (PTPRC) expression. Erythrocytes were removed by eliminating any barcode with more than 1% of hemoglobin expression. The residual barcodes were considered healthy CTCs for further analysis. The patients having less than 5 CTCs were excluded. All ALDH isoforms were pooled together as PanALDH genes for analysis. The gene expression was log-normalized for principal component analysis (PCA) and t-distributed stochastic neighbor embedding (tSNE) methods. Cell clustering was performed based on the shared nearest neighbor (SNN) method. For pathway analysis, 1000 significant top-ranked genes were identified using Seurat. Then, the significantly differential genes were applied to Enrichr (http://amp.pharm.mssm.edu/Enrichr/), and the pathway dataset of NCI-Nature 2016 was used.

### Reporting summary

Further information on research design is available in the Nature Research Reporting Summary linked to this article.

## Supplementary information

Supplementary Information

Reporting Summary

Source Data

## Data Availability

The CTC sequencing data presented in this paper have been deposited in the Sequence Read Archive (SRA) under BioProject accession number PRJNA471754 and BioSample accession numbers SAMN09217431, SAMN09217432, SAMN09217433, SAMN09217434, SAMN09217435, SAMN09217436, SAMN09217437, SAMN09217438, SAMN09217439, SAMN09217440, SAMN09217441, SAMN09217442, SAMN09217443, SAMN09217444, SAMN09217445, SAMN09217446, SAMN09217447, SAMN09217448, SAMN09217449, SAMN09217450, SAMN09217451, and SAMN09217452. Source data are available in the Source Data file. All other data are available from the authors upon reasonable request.
