# Supplementary material for: Hydro-Seq enables contamination-free high-throughput single-cell RNA-sequencing for circulating tumor cells
Source: Nat Commun. 2019 May 15;10:2163. doi: 10.1038/s41467-019-10122-2 (PMC6520360; doi:10.1038/s41467-019-10122-2)
Supplement: Supplementary file 1 — Supplementary Information [file 41467_2019_10122_MOESM1_ESM.docx]

Supplementary Information
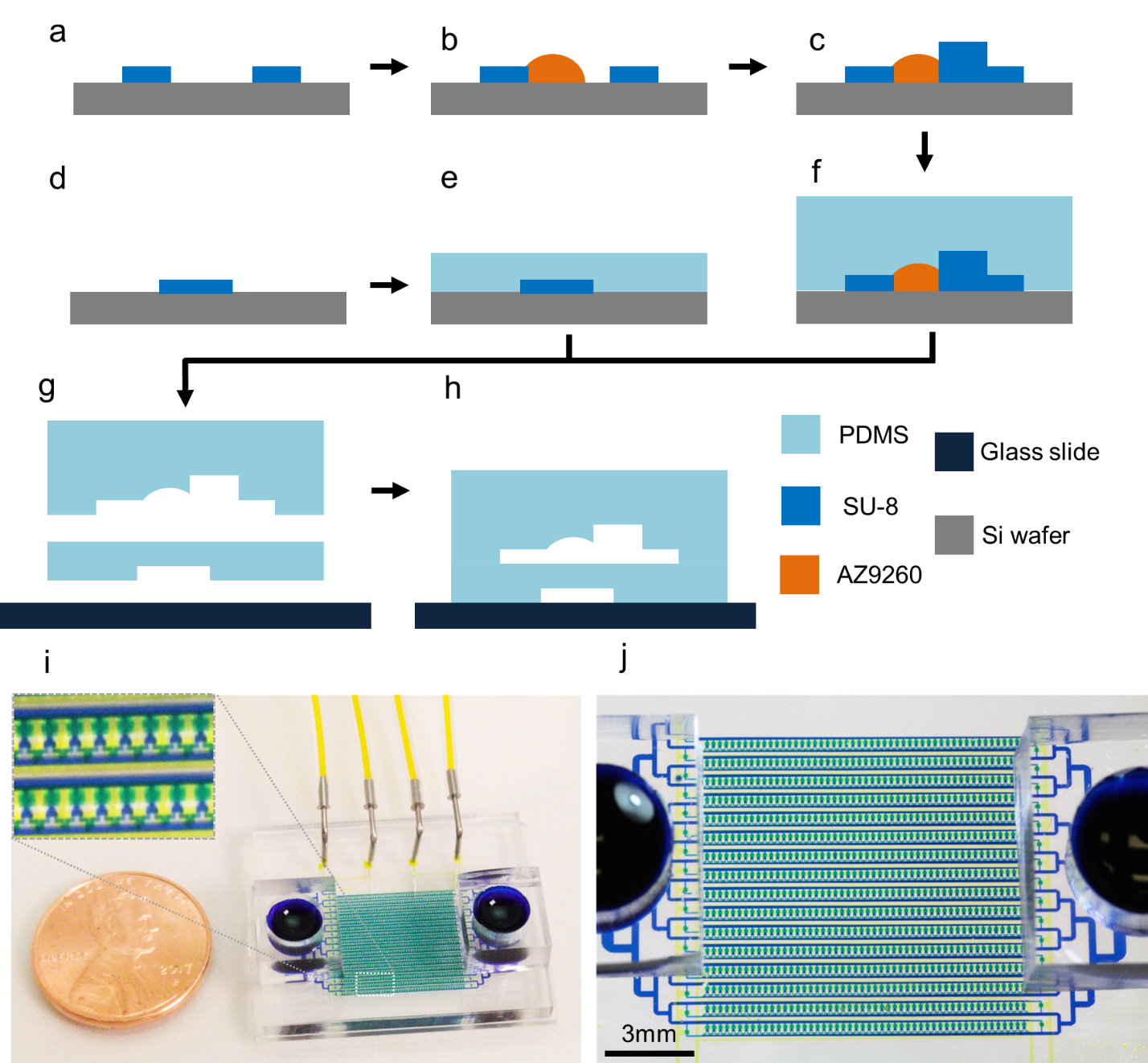


Supplementary Figure 1 | Multilayer fabrication of Hydro-Seq. (a) SU-8 patterned on the silicon wafer to create the mold for soft lithography fabrication. (b) After photoresist AZ-9260 patterning, the thermal flow process created a curved structure for valve sealing. (c) The flow channel mold was made of two layers of AZ-9260 and four layers of SU-8. (d) The valve control channel mold was made by one layer of SU-8 patterning. (e-f) After multilayer fabrication, self-assembled monolayer of silane coating is then applied to facilitate PDMS peeling. (e) The valve control layer made by spinning a 30 µm PDMS layer. (f) The flow layer made by pouring 2 mm PDMS on the mold for curing. (g) To assemble the layers together, the flow layer was detached from the mold. After alignment and surface plasma activation, the flow layer was bonded to the control channel. (h) The fabrication completed by attaching the PDMS layers to a glass substrate. (i) Photograph of the fabricated device with a US penny. Four valve control channels are connected to the device for valve manipulation. (j) Photograph of the high-density chamber array.

*
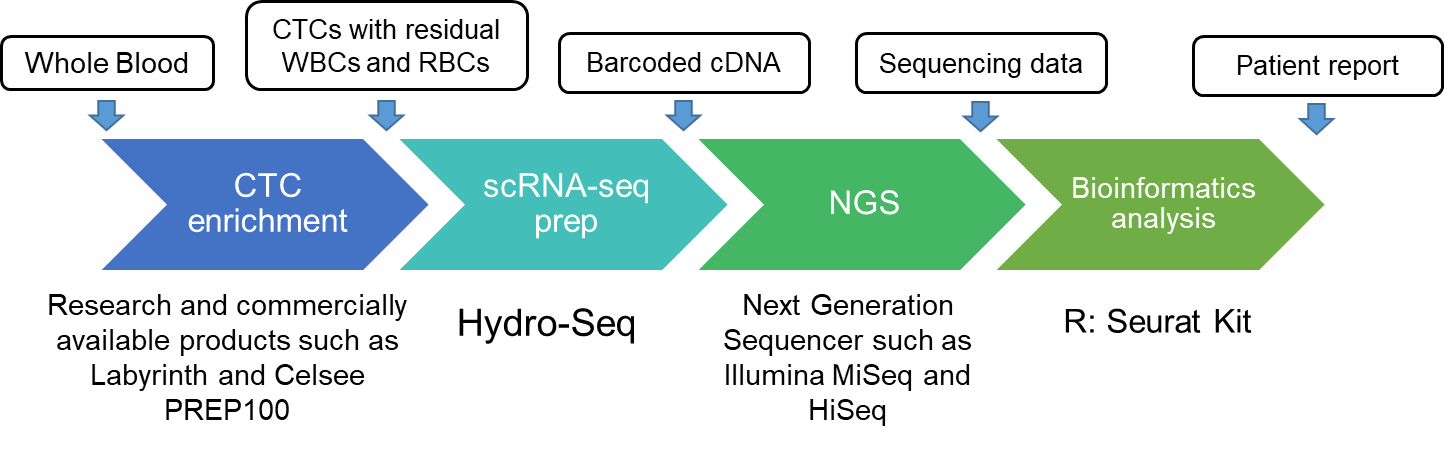
* Supplementary Figure 2 | Process flow for CTC scRNA-seq. Hydro-Seq bridges the current gap between CTC enrichment and next generation sequencing to enable single CTC RNA-seq.


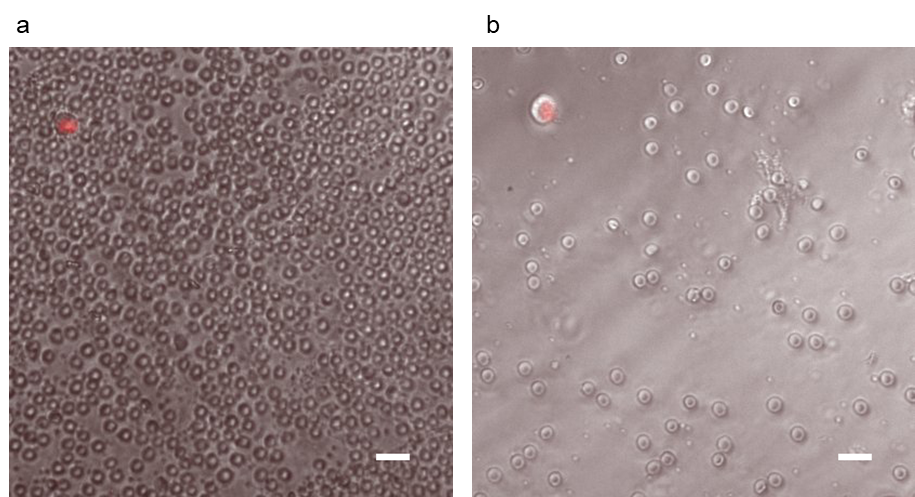


Supplementary Figure 3 | Blood sample with spike-in red fluorescent cancer cells after one (a) and two runs (b) of enrichment by Celsee chip. (scale bar: 20 µm)


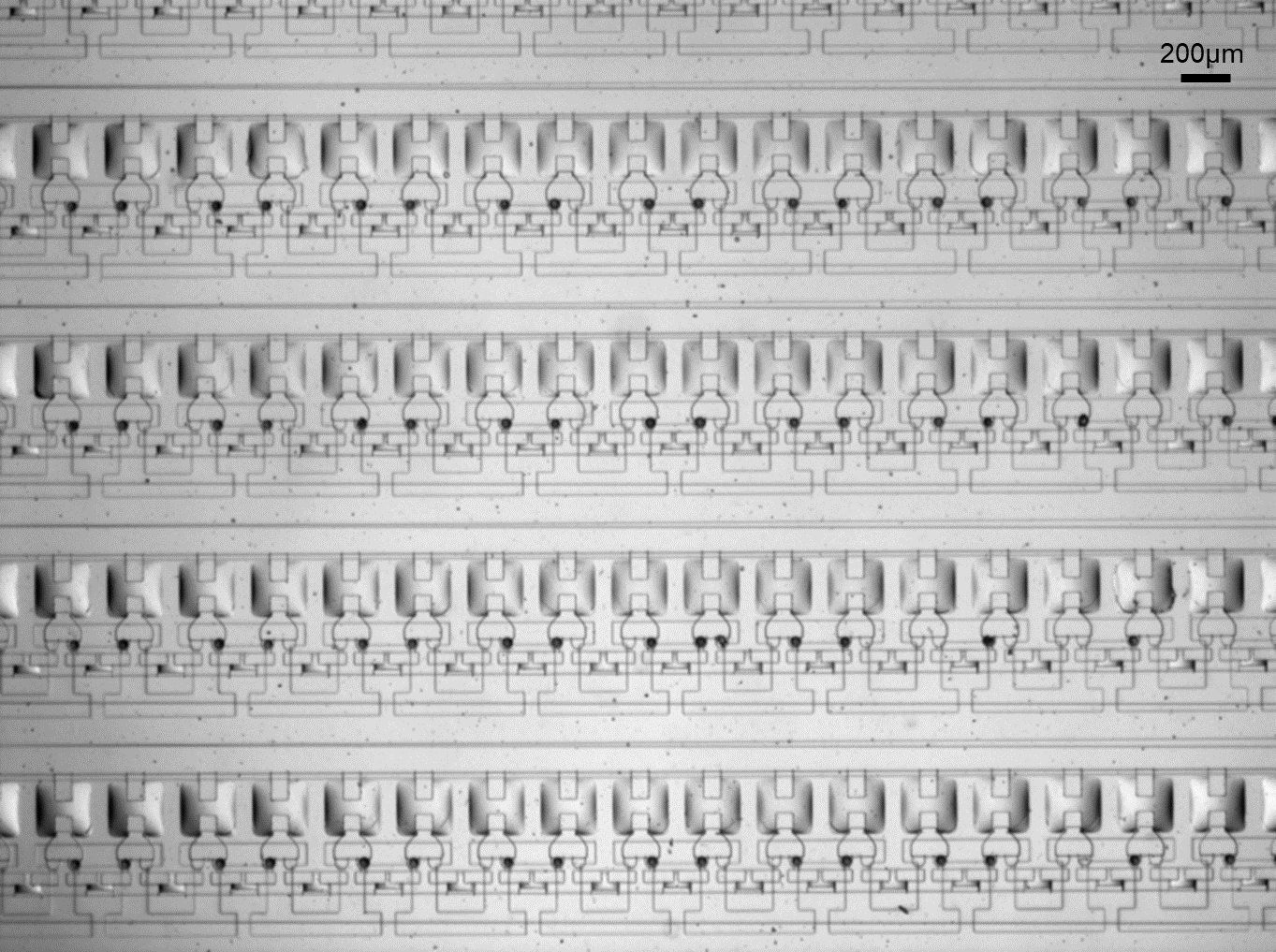


Supplementary Figure 4 | Device image after bead-cell pairing shows no adhesion of cell/bead on PDMS channels. (scale bar: 200 µm)


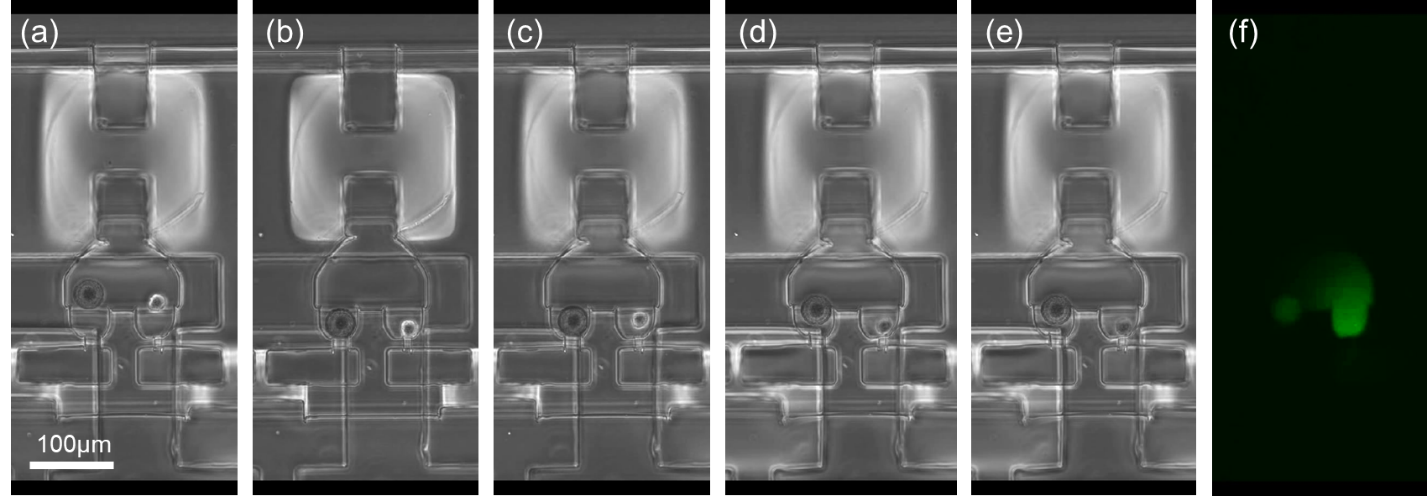


Supplementary Figure 5 | Cell lysis in a Hydro-Seq chamber. (a) Before lysis, the cell and bead are paired in a chamber and all the valves are closed. The lysis buffer is loaded to the branch channel. (b) As all the valves are opened, the laminar flow of lysis buffer pushes the bead and cell back to the capture site, and the lysis buffer flows into the chamber. As the cell seals the capture channel, the cell remains intact in the dead volume in the pocket. (c) After closing all the valves, the valve closure operation creates turbulence flow in the chamber, exposing the cell to the lysis buffer. (d-e) The cell is lysed within 30 seconds. (f) To validate cell lysis, we used a cell with green fluorescent protein (GFP). After lysis buffer was introduced, cellular contents were released showing the GFP highlighted by fluorescent imaging. (MDA-MB-231 GFP cells used in the experiment.)


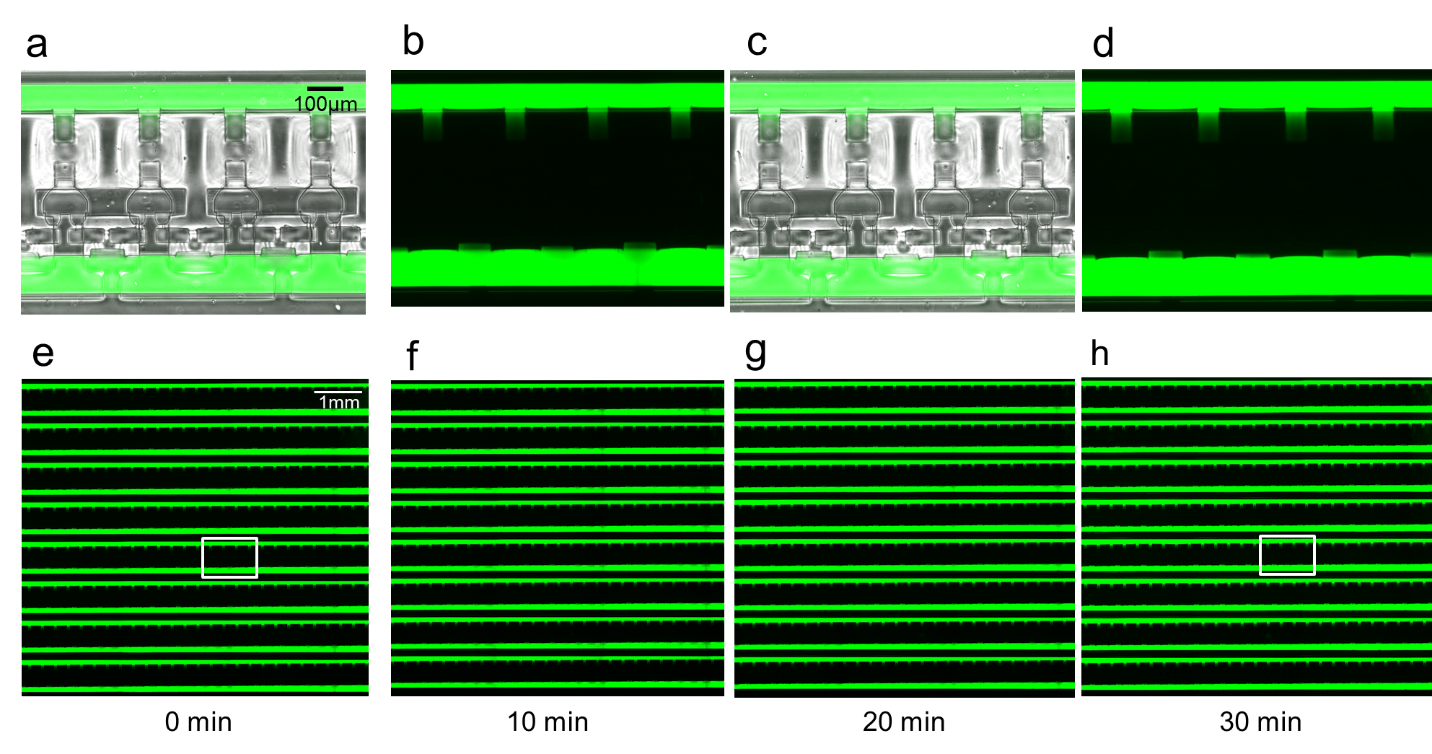


Supplementary Figure 6 | To ensure high quality single-cell analysis and prevent cross-contamination between chambers, a leakage test of valves was conducted for 30 minutes, which is the time required for incubation of mRNAs to be captured on beads. (a) Microscopic image overlapped with bright field and fluorescent images showing the parallel chambers isolated by closing the pneumatic valves at 0 minute after introducing the fluorescent dye to the chip. (b) The corresponding fluorescent image of the channel shown in (a). (c) Microscopic image overlapped with bright field and fluorescent images at 30 minutes after introducing the fluorescent dye. (d) The corresponding fluorescent image of the channel in (c). (e-h) Images highlighting the chamber isolation at 0 minute (e), 10 minutes (f), 20 minutes (g), and 30 minutes (h) after introducing the fluorescent dye. The white box in (e) is shown in (a), and the box in (h) is shown in (c).


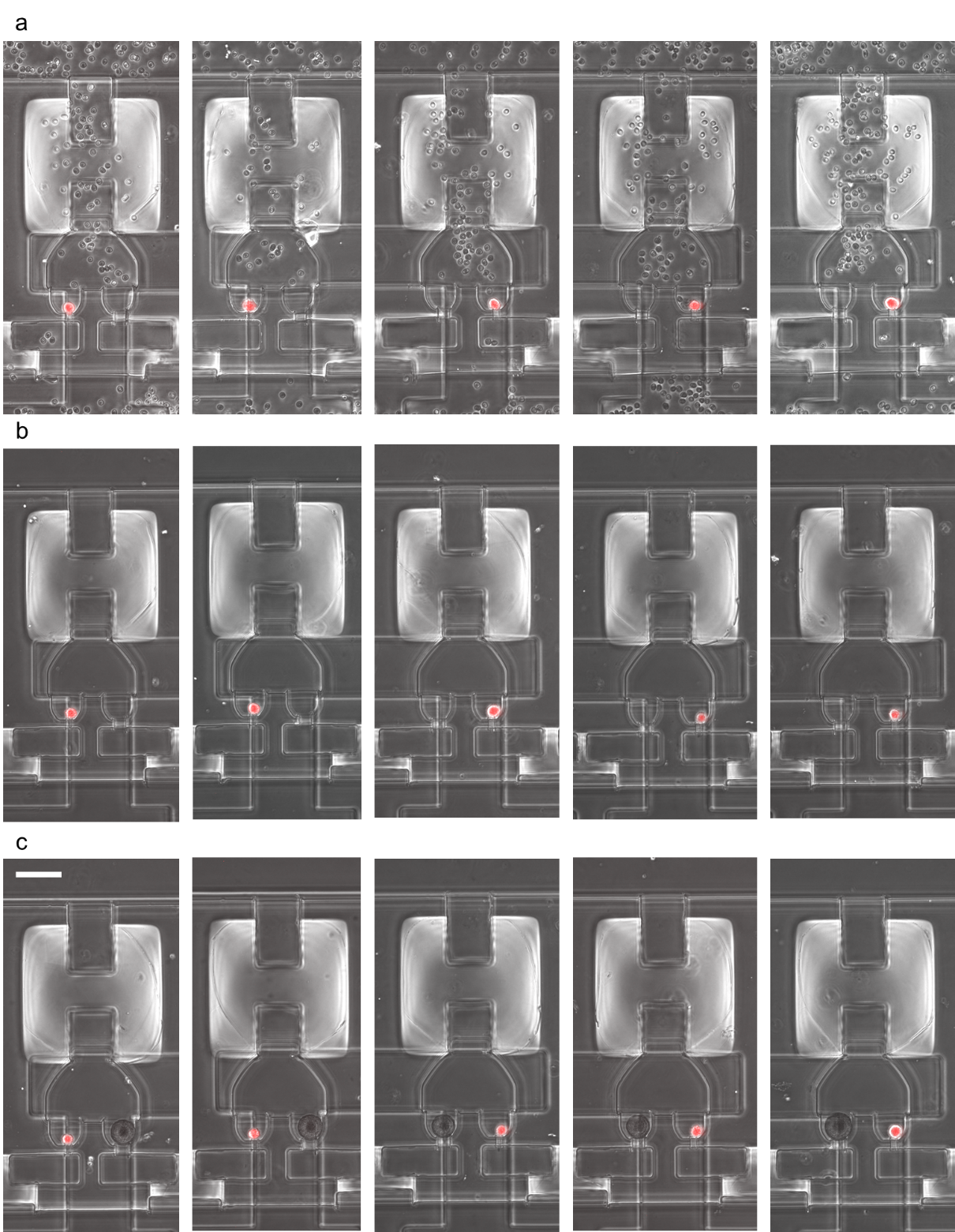


Supplementary Figure 7 | Single CTC isolation and barcoded bead pairing process. (a) The cell loading process highlights the enriched CTC samples with residual blood cells and debris in the chambers. (b) The washing step removes the background blood cells and debris to achieve contamination-free RNA-sequencing. (c) The barcoded beads are introduced to the chamber to pair with single cells. (Scale bar: 65 µm)


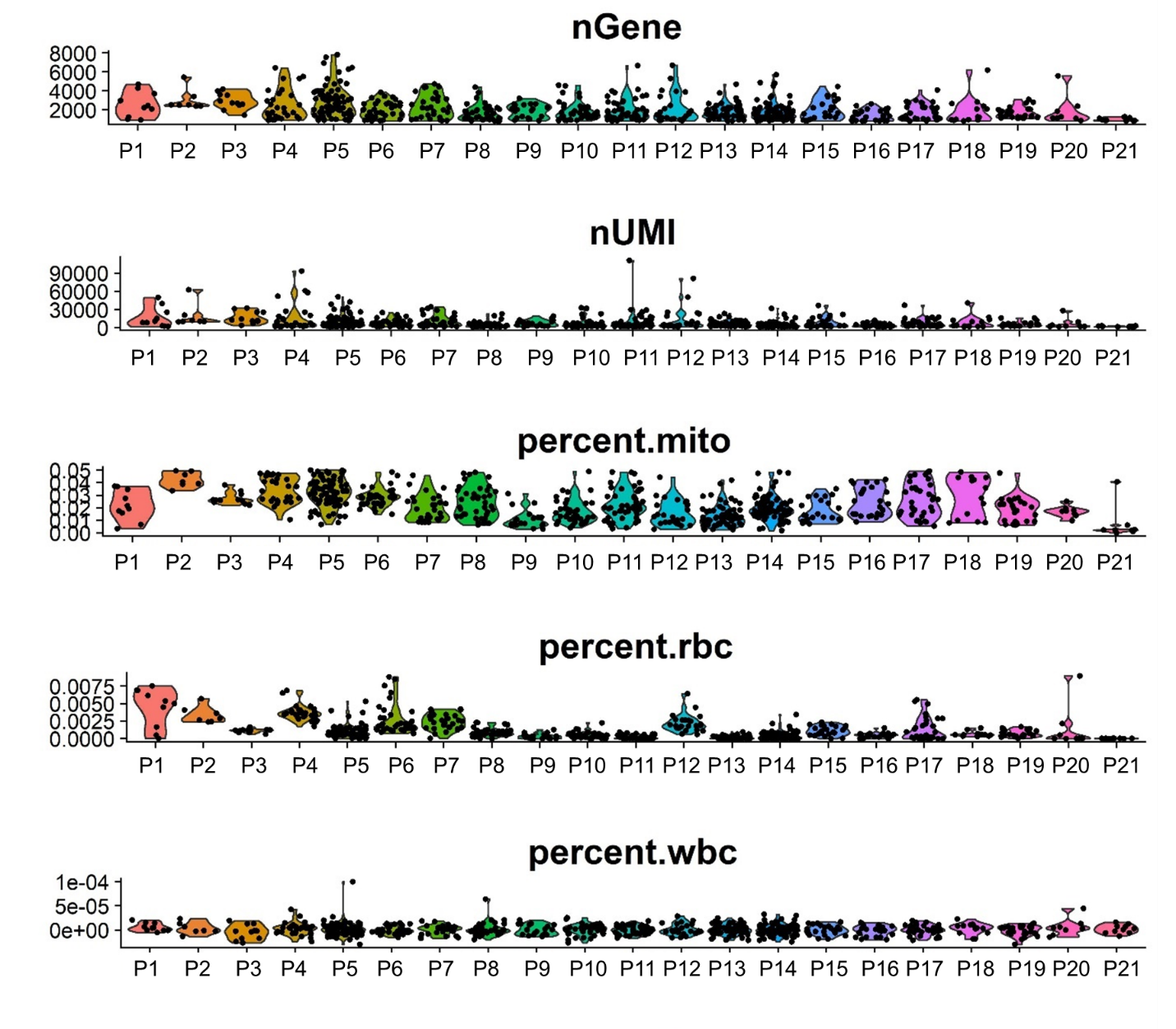


Supplementary Figure 8 | The number of genes (nGENE), the number of transcripts (nUMI), the percentage of mitochondrial genes (percent.mito), the percentage of hemoglobin genes (percent.rbc), and the percentage of CD45 gene (percent.wbc) of each patient sample. Each dot represents a CTC, and each column represents a patient sample.


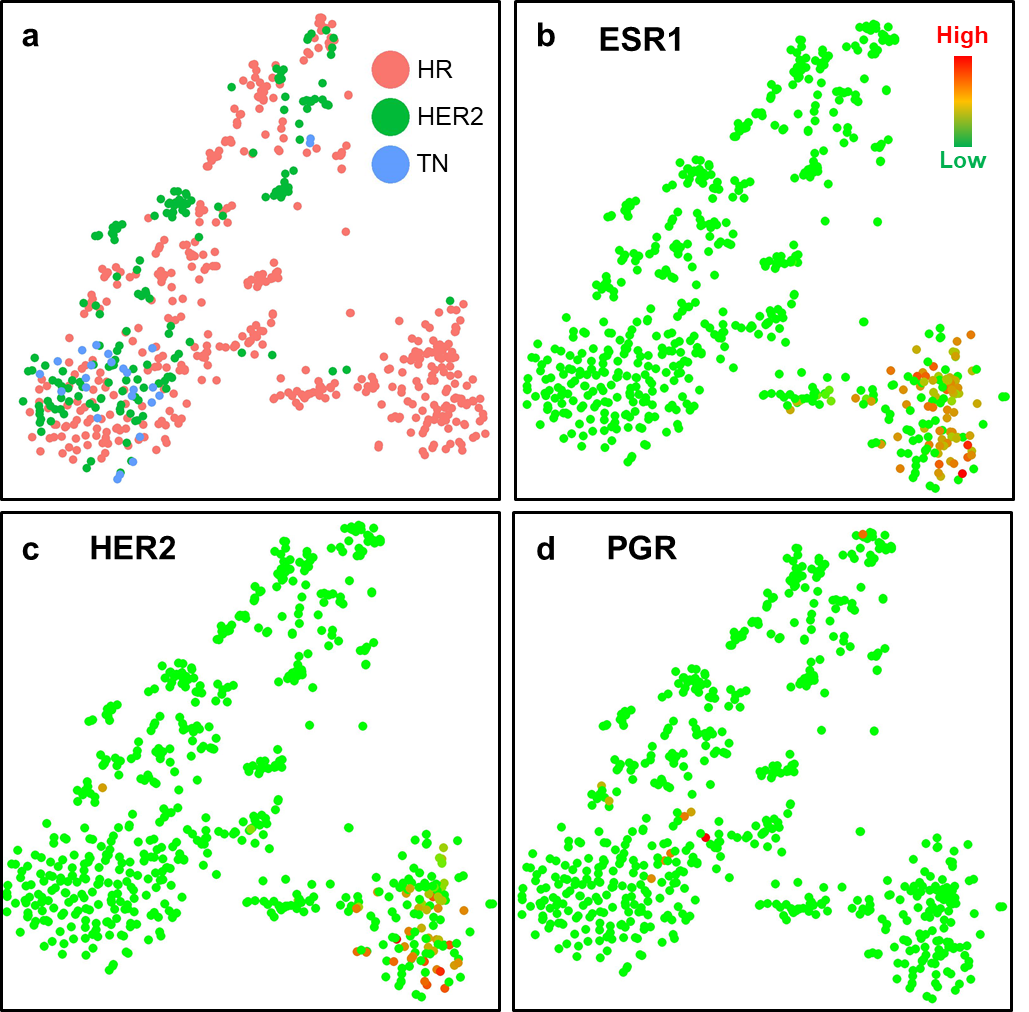


Supplementary Figure 9 | The comparison of CTCs between different molecular subtypes of primary tumor. (a) The tSNE plot of 666 CTCs from 21 patient samples. Red dots represent CTCs from hormone (estrogen or progesterone) receptor (HR) positive patients, green dots represent CTCs from HER2 positive patients, and blue dots represent CTCs from triple negative (TN) patients. (b-d) The expression of critical clinical markers: (b) estrogen receptor (ESR1), (c) human epidermal growth factor receptor 2 (HER2/Erbb2), and (d) progesterone receptor (PGR). Each dot represents one CTC. Green color represents the lowest expression, and red color represents the highest expression. The expression is logarithmically normalized.


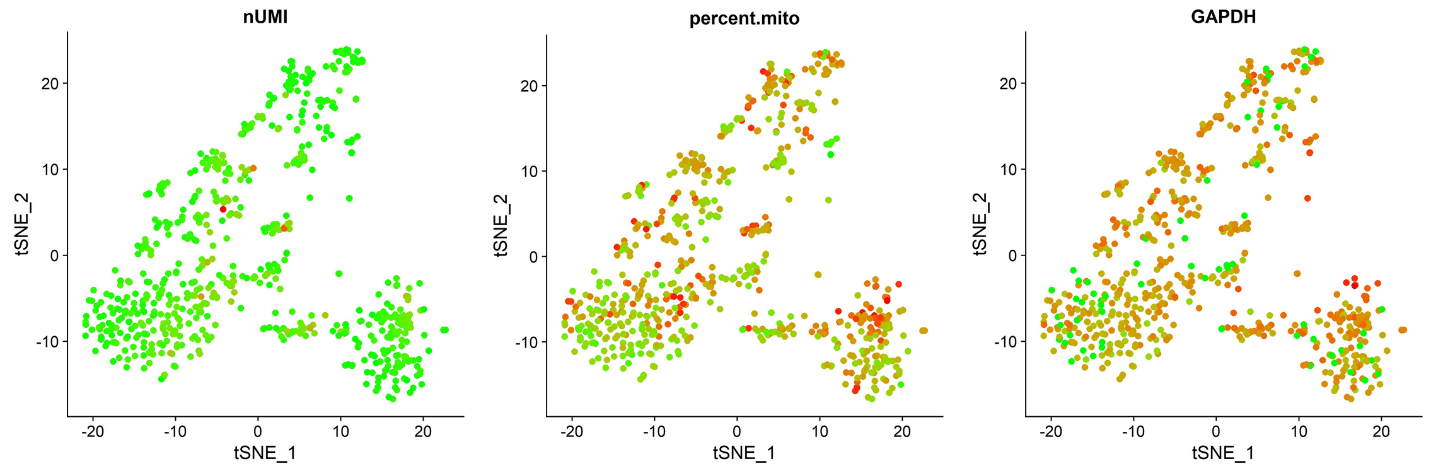
 Supplementary Figure 10 | The number of transcripts (UMI), percentage of mitochondrial genes (percent.mito), and the expression of housekeeping gene GAPDH detected per cell. Each dot represents one CTC. Green color represents the lowest expression, and red color represents the highest expression. The expression is logarithmically normalized. 666 CTCs from 21 patient samples were plotted based on tSNE clustering method. The plot verifies that the separation of clusters is not biased by number of transcripts and cell viability.


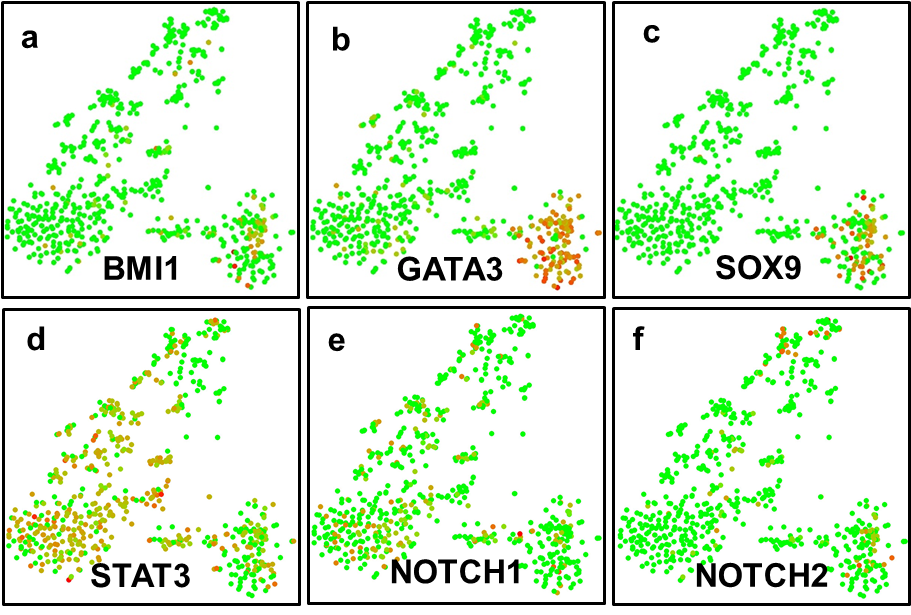


Supplementary Figure 11 | Gene expression of breast CTCs. The expression of cancer-stem-like cell (CSC) markers: (a) Polycomb complex protein BMI-1 (BMI1), (b) GATA3 transcription factor (GATA3), (c) Transcription factor SOX-9 (SOX9), (d) Signal transducer and activator of transcription 3 (STAT3), (e) Notch homolog 1 (NOTCH1), and (f) Notch homolog 2 (NOTCH2). Each dot represents one CTC. Green color represents the lowest expression, and red color represents the highest expression. The expression is logarithmically normalized. 666 CTCs from 21 patient samples were plotted based on tSNE clustering method.

Supplementary Table 1 | Cell capturing and pairing efficiency of ~10-cell spike-in tests.

|  | Exp1 | Exp2 | Exp3 | Avg. # of cells | Percentage |
| --- | --- | --- | --- | --- | --- |
| # of spiked cells* | 13 | 14 | 17 | 14.67±2.08 | - |
| # of cells after loading | 9 | 14 | 18 | 13.67±4.51 | 93.16±30.74% |
| # of cells after washing | 9 | 10 | 16 | 11.67±3.79 | 79.53±25.81% |
| # of pairs of cell/bead | 9 | 8 | 14 | 10.33±3.21 | 70.44±21.91% |

*We counted the number of spiked cells before loading to the Hydro-Seq chip by dispensing the same volume of cell suspension on 384 well plate three independent times.

Supplementary Table 2 | The patient molecular subtypes of each sample.

| Sample | Patient Subtypes | Number of CTCs | CTC Enrichment Methods |
| --- | --- | --- | --- |
| P1 | HR+ | 10 | Labyrinth |
| P2 | HR+ | 7 | Labyrinth |
| P3 | HR+ | 11 | Labyrinth |
| P4 | HR+ | 29 | Labyrinth |
| P5 | HR+ | 78 | Labyrinth |
| P6 | HER2+ | 36 | Labyrinth |
| P7 | HR+ | 29 | Labyrinth |
| P8 | HR+ | 40 | Labyrinth |
| P9 | HER2+ | 20 | Celsee |
| P10 | HER2+ | 42 | Celsee |
| P11 | HR+ | 51 | Celsee |
| P12 | HR+ | 24 | Celsee |
| P13 | HR+ | 65 | Celsee |
| P14 | HR+ | 83 | Celsee |
| P15 | HR+ | 24 | Celsee |
| P16 | TN | 25 | Celsee |
| P17 | HER2+ | 35 | Celsee |
| P18 | HER2+ | 14 | Celsee |
| P19 | HER2+ | 25 | Celsee |
| P20 | HR+ | 8 | Celsee |
| P21 | HR+ | 10 | Celsee |

Supplementary Table 3 | Technology benchmarking for scRNA-seq.

| Mechanism | Name | Reagent Operation | Throughput | Cell capture efficiency | Washing capability |
| --- | --- | --- | --- | --- | --- |
| Manual Picking[1] | Smart-seq | Volume filling | ~10 | High | N.A. |
| Robotic Picking[2] | N.A. | Volume filling | ~100 | High | N.A. |
| Droplet | Drop-seq[3] | Bead pairing | ~1,000/assay | < 5% | No |
| Droplet | 10x Genomics[3] | Bead pairing | ~1,000/assay | ~ 50% | No |
| Micro-well | Seq-Well[4] | Bead pairing | ~10,000/chip | ~ 35% | No |
| Micro-well | CytoSeq[5] | Bead pairing | ~3,000/chip | ~ 15% | No |
| Hydrodynamics | C1[6] | Volume filing | ~96/chip | ~ 10% | No |
| Hydrodynamics | Hydro-Seq | Bead pairing | ~800/chip | ~ 70% | Yes |

Supplementary Methods

Hydro-Seq Protocol

**A. CTCs enrichment (Celsee protocol)**

**B. Device preparation**

1. Heat the device at 150^o^C for 25 minutes to deactivate any enzyme activities on the chip. (**While waiting**, use ethanol and RNase Zap to wipe and clean the desiccator.)

2. Place the device in a desiccator with vacuum pressure for 15 minutes.
(**While waiting**, organize the hood and use ethanol and RNase Zap to wipe and clean the hood.)

3. Prime the device using a 5% (w/w) PEO-terminated triblock polymer (Pluronic® F108, BASF) in DI-water and sanitize the device using UV radiation for 15 mins.
*(****NOTE:*** *To prime the device, first put 100uL F108 into the inlet, then withdraw 50uL using pipette from the outlet, finally fill both the inlet and the outlet with F108. Also, cover the tubing ports with F108.)* 
(**While waiting,** use ethanol and RNase to wipe and clean the microscope and computer. Open the gas regulator valve, with the left meter reading being about 20. Open valve control.vi.)

4. Place 100uL PBS in the inlet.

5. Check the tubing by flashing the controls until liquid shows at the end. Connect tubing to designated ports. Examine the device using a microscope to ensure good valve operation.

6. Wipe the scissor with ethanol and RNase Zap. Cut a tubing tapered to make it easier to insert into the outlet.

7. Take a 1mL syringe and fill it with 500uL of PBS. Install the needle and the tubing. Mount the syringe onto the pump and squeeze out PBS to the open end of the tubing.

8. Connect tubing from the outlet to syringe.

9. Withdraw 65uL at 50uL/min to wash the channel with PBS.

**C. Cell loading**

10. Close the bead valve and wash channel valves (16, 19, 20)

11. Load the cell suspension with a pipette tip. Start the syringe pump to withdraw cell suspension at 10uL/min. (***CAUTION:*** *Pay attention to how the water level inside the pipette. Make sure air/bubbles don’t get into the device.)*

12. Remove the pipette tip after loading. Add 150uL PBS to the inlet. Withdraw at 10uL/min until the channels are clear of residual cells.

13. Keep withdrawing. Add PBS if needed. Open the bead valve (20). Change the flow rate to 50uL/min.

14. Keep withdrawing. Open all the valves. Stop until the channels are clear.

15. Close main valve (18) and then the bead valve (20). Remove the tubing. Wash the channels back and forth with pipette until the inlet and the outlet are almost clear.

16. Only open main valve (18). Fill the outlet with 150uL PBS. Withdraw the 100uL PBS from outlet to inlet to retrieve the cells back to the pipette. Leave the pipette tip in the inlet. (***CAUTION:*** *Procced quickly to the next step.*)

17. Install the tubing back to the outlet and withdraw from inlet to outlet with 10uL until emptying the pipette tip.

18. Remove the pipette tip after loading. Load the inlet with 150uL PBS. Withdraw at 10uL/min until the channels are clear of residual cells.

19. Keep withdrawing. Add PBS if needed. Open the bead valve (20). Change the flow rate to 50uL/min.

20. Keep withdrawing. Open all the valves. Stop until the channels are clear.

**D. Bead loading**

24. Adjust the syringe pump to flow rate: 50uL/min and flow volume 100uL (Checkpoint: 75uL)

25. Spin down 100uL bead solution. Remove supernatant. Add 100uL PBS.

26. Only close left wash channel valve (16).

27. Load the 100uL PBS and beads into the inlet. Add more PBS (~50uL) until the inlet is full. Start the syringe withdraw and pipette to mix during loading.
*(****CAUTION:*** *Don’t introduce bubbles while pipetting. Always make sure that there is adequate liquid in the inlet. Stop and check after 75uL is flowed to make sure there is enough liquid in the inlet.)*

28. Stop the flow if the majority of chambers capture one bead.

29. Close the main valve and then the bead valve (20, 18). Open the washing channel valves.

**E. Cell lysis**

30. Prepare 100uL lysis mix

***Lysis mix recipe (makes 100uL)****:*
95uL Lysis Buffer 1x on rack ***CAUTION:*** *Not 5x buffer*
5uL DTT on rack

31. Remove the remaining PBS in the inlet and load 100uL lysis mix to the inlet.

32. Install vacuum pump to the blue port of the tubing system, and activate the pump.

33. Move the view of the microscope to the top of the chip.

34. Set the syringe pump to withdraw 80uL at 50uL. **CHECKPOINT:** After withdrawing 15uL lysis buffer, proceed the next steps.

35. Open the bead valve (20).

36. Open the main valve (18).

37. Close the main valve (18) and then bead valve (20) after the lysis mix enter the chamber.

38. Move the bead to the left pocket by gravity and incubate for 5 mins.

39. (**While waiting**) Remove the vacuum pump.

40. (**While waiting**) Unfreeze RT mix ingredients which are in the freezer (see below) for 10 mins.

41. Move the bead to the right pocket by gravity and incubate for 5 mins.

42. Move the bead to the left pocket by gravity and incubate for 5 mins.

43. (**While waiting)** Prepare RT mix (***CAUTION:*** *Don’t add Maxima H- RTase for now.*)

***RT mix recipe (makes 100uL)****: (can be prepared in advance without RTase)*
37.5uL H_2_O from fridge
20uL Maxima 5x RT Buffer from freezer
20uL 20% Ficoll PM-400 from fridge
10uL 10mM dNTPs (Clontech) from freezer
2.5uL RNase Inhibitor (Lucigen) form freezer
5uL 50uM Template Switch Oligo form freezer, small tube
~~5uL Maxima H- RTase from freezer, tube with red cap~~

44. Move the bead to the right pocket by gravity and incubate for 5 mins.

45. Reload inlet with 100uL PBS and withdraw 20uL at 50uL/min to remove the lysis buffer in the channels.

**F. Bead retrieval**

46. After incubation, make sure the level in inlet is higher than that in the outlet, open the valve and remove all valve control tubing.

47. Shut the gas regulator valve. Both of the meters should go to 0.

48. Remove the all the liquid in the outlet. Then, remove all in the inlet. (***CAUTION:*** The order matters.)

49. Move the device to the hood.

50. Add 200uL PBS to the outlet.

51. Withdraw 100uL from the outlet to inlet and dispense the solution in a tube.

52. Add 100uL PBS to the outlet.

53. Withdraw 100uL from the outlet to inlet and dispense the solution in the same tube.

54. The tube now contains 200uL PBS with 99.9% of the beads from the device.

**G. Reverse transcription**

55. Spin down and remove supernatant. Add 100uL PBS.

56. Spin down and remove supernatant. Add 100uL 5xRT Buffer.

57. Spin down and remove supernatant.

58. Finish the RT mix by adding 5uL Maxima H- RTase.

59. Add 100uL RT mix to the beads. Mix by pipetting up and down.

60. Incubate at room temperature for 30 minutes with rotation.

61. Incubate at 42^o^C for 90 minutes with rotation. Set heater to 37^o^C after this.

62. Wash the beads once with 100uL TE-SDS (on workbench), 3 times with 100uL TE-TW (on workbench) *(****STOPPING POINT****: Beads can be stored at 4^o^C in TE-TW.* ***CAUTION:*** *Check the gas regulator and cover the microscope before leaving.)*, and then if proceeding to exonuclease I treatment wash once more with 100uL 10mM Tris pH 8.0.

63. (**While waiting**) Unfreeze Exonuclease mix ingredients which are in the freezer (see below) for 10 mins.

**H. Exonuclease I treatment**

64. Prepare Exonuclease mix *(****NOTE:*** *The reagents need to be unfrozen for 10 mins before use)*

***Exonuclease mix recipe (makes 100uL):***
10uL 10x Exo I Buffer in the freezer
85uL H_2_O
5uL Exo I in the freezer

65. After washing once with 100uL 10mM Tris pH 8.0, re-suspend in 100uL of exonuclease mix.

66. Incubate at 37^o^C for 45 minutes with rotation.

67. Wash the beads once with 100uL TE-SDS, 3 times with 100uL TE-TW *(****STOPPING POINT****: Beads can be stored at 4^o^C in TE-TW.* ***CAUTION:*** *Check the gas regulator and cover the microscope before leaving.)*, and then if proceeding to PCR, wash once more with 100uL H_2_O (good DI water).

**I. Preparing for PCR**

68. After washing once with 100uL H_2_O (good DI water), spin down, remove supernatant, and add another 100uL H_2_O (good DI water).

69. Spin down the tubes, and add the following PCR mix (per tube):

24.6uL H_2_O***CAUTION****: Add less considering how much liquid left in the tube*
0.4uL 100uM SMART PCR PRIMER in the freezer, in a box
25uL 2x Kapa HiFi Hotstart Readymix in the freezer, in a box

70. Mix well and proceed to PCR.

**J. PCR program**

71. Check PCR program before use.

72. PCR cycle.

95^o^C 3 minutes

4 cycles of:

98^o^C 20 s
65^o^C 45 s
72^o^C 3 min

11 cycles of:

98^o^C 20 s
67^o^C 20 s
72^o^C 3 min

72^o^C 5 min

4^o^C forever

73. (**While waiting**) Unfreeze AMPure XP which are in the freezer.

**K. DNA purification**

74. (**While waiting**) Make 300uL 70% Ethanol for each sample, plus extra.

Ethanol is in the flammable cabin outside the room.

75. Shake AMPure XP. Add 30uL (0.6x reaction volume) AMPure XP to the tube. Pipette up and down for 10+ times. Put AMPure XP back into the fridge. Wait for 5 mins.

76. Put the magnet under the holding board, and place the tube on the board for 2 mins.

77. Place the magnet next to the tube, take supernatant *(****CAUTION:*** *leave 5uL in the tube)*.

78. Add 100uL ethanol *(****CAUTION:*** *While adding, try not to touch the beads)*, wait for 30 secs with the magnet under the tube.

79. Take ALL supernatant.

80. **Repeat** ethanol wash for total 3 times.

81. Add 20uL H_2_O (good DI water). Pipette up and down for 10+ times. Wait for 2 mins. (**While waiting**, mark new tubes.)

82. Put the magnet under the tube for 1 min.

83. Place the magnet beside the tube, take 20uL supernatant, and put into the marked tube.

84. Move 5uL to another tube.

85. Store the sample in the freezer for test. *(****CAUTION:*** *Check the gas regulator and cover the microscope before leaving.)*

Supplementary Reference

[1] Miyamoto, D. T. *et al.* RNA-Seq of single prostate CTCs implicates noncanonical Wnt signaling in antiandrogen resistance. *Science (80-. ).* **349,** 1351–1356 (2015).

[2] Lohr, J.G. *et al.* Whole-exome sequencing of circulating tumor cells provides a window into metastatic prostate cancer. *Nat Biotechnol.* **32**(5), 479-84 (2014).

[3] Zhang, X. *et al.* Comparative Analysis of Droplet-Based Ultra-High-Throughput Single-Cell RNA-Seq Systems. *Mol Cell.* **73**(1), 130-142.e5 (2019).

[4] Gierahn, T. M. *et al.* Seq-Well: portable, low-cost RNA sequencing of single cells at high throughput. *Nat. Methods* **14,** 395–398 (2017).

[5] Fan, H. C., Fu, G. K. & Fodor, S. P. A. Combinatorial labeling of single cells for gene expression cytometry. *Science (80-. ).* **347**(6222), 1258367, (2015).

[6] Tung, P.-Y. *et al.* Batch effects and the effective design of single-cell gene expression studies. Sci Rep. **7**, 39921 (2017).
