# Supplementary material for: Hydro-Seq enables contamination-free high-throughput single-cell RNA-sequencing for circulating tumor cells
Source: Nat Commun. 2019 May 15;10:2163. doi: 10.1038/s41467-019-10122-2 (PMC6520360; doi:10.1038/s41467-019-10122-2)
Supplement: Supplementary file 2 — Reporting Summary [file 41467_2019_10122_MOESM2_ESM.pdf]

nature research

Corresponding author(s): *Double-blind peer review submissions: write DBPR and your manuscript number here instead of author names.*

Reporting Summary

Nature Research wishes to improve the reproducibility of the work that we publish. This form provides structure for consistency and transparency in reporting. For further information on Nature Research policies, see Authors & Referees and the Editorial Policy Checklist.

Statistical parameters

When statistical analyses are reported, confirm that the following items are present in the relevant location (e.g. figure legend, table legend, main text, or Methods section).

n/a	Confirmed	
☐	☒	The exact sample size (*n*) for each experimental group/condition, given as a discrete number and unit of measurement
☐	☒	An indication of whether measurements were taken from distinct samples or whether the same sample was measured repeatedly
☐	☒	The statistical test(s) used AND whether they are one- or two-sided<br>*Only common tests should be described solely by name; describe more complex techniques in the Methods section.*
☐	☒	A description of all covariates tested
☐	☒	A description of any assumptions or corrections, such as tests of normality and adjustment for multiple comparisons
☐	☒	A full description of the statistics including central tendency (e.g. means) or other basic estimates (e.g. regression coefficient) AND variation (e.g. standard deviation) or associated estimates of uncertainty (e.g. confidence intervals)
☒	☐	For null hypothesis testing, the test statistic (e.g. *F*, *t*, *r*) with confidence intervals, effect sizes, degrees of freedom and *P* value noted<br>*Give P values as exact values whenever suitable.*
☒	☐	For Bayesian analysis, information on the choice of priors and Markov chain Monte Carlo settings
☒	☐	For hierarchical and complex designs, identification of the appropriate level for tests and full reporting of outcomes
☒	☐	Estimates of effect sizes (e.g. Cohen's *d*, Pearson's *r*), indicating how they were calculated
☒	☐	Clearly defined error bars<br>*State explicitly what error bars represent (e.g. SD, SE, CI)*

Our web collection on statistics for biologists may be useful.

Software and code

Policy information about availability of computer code

Data collection	NIS-Elements Basic Research for microscopy imaging
Data analysis	Seurat v2.0 and R 3.3 for single-cell transcriptome data analysis

For manuscripts utilizing custom algorithms or software that are central to the research but not yet described in published literature, software must be made available to editors/reviewers upon request. We strongly encourage code deposition in a community repository (e.g. GitHub). See the Nature Research guidelines for submitting code & software for further information.

Data

Policy information about availability of data

All manuscripts must include a data availability statement. This statement should provide the following information, where applicable:

- Accession codes, unique identifiers, or web links for publicly available datasets
- A list of figures that have associated raw data
- A description of any restrictions on data availability

The CTC sequencing data (Fig.2-5) presented in this paper have been deposited in the Sequence Read Archive (SRA) under BioProject accession number PRJNA471754.

Field-specific reporting

Please select the best fit for your research. If you are not sure, read the appropriate sections before making your selection.

☒ Life sciences ☐ Behavioural & social sciences ☐ Ecological, evolutionary & environmental sciences

For a reference copy of the document with all sections, see nature.com/authors/policies/ReportingSummary-flat.pdf

Life sciences study design

All studies must disclose on these points even when the disclosure is negative.

Sample size	We used 21 patient samples for the validation of our engineering tool.
Data exclusions	If a sample has less than 5 CTCs isolated, we discard the sample.
Replication	We used a mouse cell line (3T3) and a human cell line (HEK) for reproducibility test in Fig.2. We also collected two tubes of blood from the same patient at the same time to show reproducibility in Fig.3.
Randomization	N/A
Blinding	N/A

Reporting for specific materials, systems and methods

Materials & experimental systems

n/a	Involved in the study
☒	☐ Unique biological materials
☐	☒ Antibodies
☐	☒ Eukaryotic cell lines
☒	☐ Palaeontology
☒	☐ Animals and other organisms
☐	☒ Human research participants

Methods

n/a	Involved in the study
☒	☐ ChIP-seq
☒	☐ Flow cytometry
☒	☐ MRI-based neuroimaging

Antibodies

Antibodies used	Mouse Anti-Human CD45 (clone HI30) BD Biosciences Cat # 555480.
Validation	The antibody was validated by vendor.

Eukaryotic cell lines

Policy information about cell lines

Cell line source(s)	MDA-MB-231 cells were obtained from Dr. Gary Luker’s Lab (University of Michigan, MI, USA). MDA-MB-231 GFP cells were obtained from Dr. Celina Kleer’s Lab (University of Michigan, MI, USA). HEK293 and 3T3 cells were obtained from Dr. Max Wicha’s Lab (University of Michigan, MI, USA).
Authentication	The cell lines were authenticated.
Mycoplasma contamination	Mycoplasma contamination was done regularly and proved to be negative.
Commonly misidentified lines (See ICLAC register)	N/A

Human research participants

Policy information about studies involving human research participants

Population characteristics	Study subjects consisted of women and men with metastatic breast cancer at any stage of treatment, with a minimum hemoglobin level to enable safe blood collection.
Recruitment	Subjects with metastatic breast cancer were recruited through the University of Michigan Breast Oncology Clinic.
